# Micronized Acellular Dermal Matrix and Synthetic Polymers: Temporal Macrophage Transition as a Mechanistic Framework

**DOI:** 10.3390/ijms27209239

**Published:** 2026-10-18

**Authors:** Zhenyi Jin, Caijun Jin, Zhiyuan Ding, Dang Truong Hai, Huizhen Ming, Suk Bae Seo, Moon-Seop Choi, Jung Hee Shim, Vo Tien Huy, Pham Ngoc Chien, Chan Yeong Heo

**Affiliations:** 1 Department of Plastic and Reconstructive Surgery, College of Medicine, Seoul National University, Seoul 03080, Republic of Koreadthai@ctump.edu.vn (D.T.H.);; 2 Department of Plastic and Reconstructive Surgery, Seoul National University Bundang Hospital, Seongnam 13620, Republic of Koreaxmylife@empas.com (J.H.S.); 3 Department of Dermatology, Seoul National University Bundang Hospital, Seongnam 13620, Republic of Korea; 4 Department of Anatomy, VNU University of Medicine and Pharmacy, Vietnam National University, Hanoi 100000, Vietnam

**Keywords:** micronized acellular dermal matrix, biodegradable polymers, macrophage switching, macrophage polarization window, extracellular matrix remodeling, injectable regenerative biomaterials

## Abstract

Injectable biodegradable polymers and micronized acellular dermal matrix (mADM) have potentially complementary regenerative properties, but how their combination shapes the host response remains poorly defined. In many systems, polymers provide persistent material-associated stimulation that may sustain macrophage-driven foreign-body responses, whereas mADM provides tissue-derived extracellular matrix (ECM) cues that support cellular infiltration, vascularization, and remodeling, with persistence dependent on processing and host-mediated replacement. This narrative review evaluates whether changes in macrophage population states could help explain potential polymer–mADM synergy. Across the reviewed polymer–ECM hybrid studies, most evidence is indirect, and temporal sampling rarely resolves sequential state changes. No included study establishes temporal coupling as a causal mechanism. We therefore propose a temporal macrophage-switching model in which both components act from implantation and their relative contributions change over time; their spatiotemporal overlap may shape macrophage population trajectories and remodeling outcomes. We further define a functional macrophage polarization window using biological landmarks rather than fixed time points. The model remains a testable hypothesis whose applicability depends on material and tissue context. Validation requires polymer-only, mADM-only, and hybrid comparisons across independent mADM lots, with systematic variation in polymer persistence, mADM processing, component ratio, spatial organization, and degradation/remodeling kinetics.

## 1. Introduction

The use of injectable biomaterials has expanded well beyond their traditional role as immediate soft-tissue fillers. They are increasingly viewed as regenerative platforms that can influence host cellular responses and support extracellular matrix (ECM) remodeling [1,2]. Among these materials, synthetic biodegradable polymers and acellular dermal matrix (ADM)-derived products have attracted considerable interest because they offer distinct and potentially complementary biological properties [3]. Polymers such as poly-L-lactic acid (PLLA) and poly-D,L-lactic acid (PDLLA) provide persistent material-associated stimulation, promoting fibroblast activation and neocollagenesis through a foreign-body response. Micronized acellular dermal matrix (mADM), by comparison, provides a naturally derived, collagen-rich ECM scaffold that supports cellular infiltration, angiogenesis, and progressive tissue remodeling [4,5,6,7]. However, each material also has clear limitations. Persistent recognition of polymer particles, together with degradation-associated stimulation, may sustain macrophage and foreign-body giant-cell (FBGC) activity and, under unfavorable conditions, contribute to fibrotic encapsulation or granulomatous foreign-body reactions [8,9,10,11]. mADM provides biologically instructive ECM cues, but its structural persistence is limited by enzymatic degradation and gradual replacement by host tissue [3,12,13]. These characteristics provide a reasonable basis for polymer–ECM hybridization, in which sustained material-associated biostimulation is combined with an ECM environment that supports cellular integration and constructive remodeling [3,14,15].

Macrophages are central regulators of the host tissue response to implanted biomaterials [9]. After implantation, recruited monocytes differentiate into macrophages that respond to the implant surface and its degradation by-products, as well as to adsorbed plasma proteins and surrounding matrix components [9,16,17]. M1-like (classically activated/pro-inflammatory) macrophages are associated with early inflammatory signaling, whereas M2-like (alternatively activated/resolving or pro-remodeling) macrophages contribute to resolution, angiogenesis, matrix turnover, and repair [9,18]. However, biomaterial-associated macrophages occupy heterogeneous and transitional states rather than discrete M1/M2 categories [9,17]. Implant outcomes may therefore depend on response magnitude, persistence, and whether early activation is followed by resolving/remodeling states [9,17,19,20]. Temporally controlled biomaterial studies further indicate that response timing can influence regeneration [20,21,22]. In our view, whether polymer–mADM combinations actually use this type of temporal macrophage regulation as a mechanism of synergy remains unresolved. In this review, we therefore use the term synergy as a testable hypothesis of functional complementarity between polymer-associated biostimulation and mADM-derived remodeling cues, rather than as evidence of demonstrated supra-additive efficacy.

Polymer-associated stimulation may persist during particle residence and degradation, depending on formulation and tissue context [11,23], whereas the accessibility and activity of mADM-derived cues evolve with cellular infiltration and matrix remodeling [24,25,26,27]. Both inputs may therefore act from implantation while changing in relative contribution over time. We propose that their magnitude, duration, and spatiotemporal overlap shape macrophage trajectories and tissue remodeling; testing this temporal macrophage-switching framework requires time-resolved polymer-only, mADM-only, and hybrid comparisons [20,21,22].

Previous reviews of decellularized extracellular matrix (dECM)/ADM and their composites have addressed material preparation, preservation of ECM composition and architecture, host interactions, and clinical translation [1,3,28]. Reviews of injectable synthetic polymers and foreign-body responses have examined material persistence and degradation, immune responses, and neocollagenesis [2,5,11]. Temporal changes in macrophage states and their influence on tissue repair have also been investigated [9,21,29]. Building on this work, the novelty of the present review lies in a testable framework specific to polymer–mADM hybrids that links the spatiotemporal overlap of polymer-associated stimulation and mADM-derived remodeling cues to macrophage trajectories and tissue-remodeling outcomes. We use this framework to evaluate evidence for its direct applicability to polymer–mADM systems and relevance to the temporal model, propose a functional macrophage polarization window, and formulate mechanistic predictions testable by varying component ratio, spatial distribution, and degradation/remodeling kinetics. Supplementary Table S1 summarizes the previously reviewed domains, unresolved questions, and the specific contribution of the present review.

## 2. Methodology

This study was designed as a mechanism-oriented narrative review rather than a systematic review or meta-analysis. PubMed, Web of Science, and Scopus were searched through 8 September 2026 using terms related to: (1) biodegradable synthetic polymers, including PLLA, PDLLA, poly(lactic-co-glycolic acid) (PLGA), polycaprolactone (PCL), and poly(L-lactide-co-ε-caprolactone) (PLCL); (2) decellularized extracellular matrix (dECM), acellular dermal matrix (ADM), micronized ADM (mADM), and tissue-derived ECM; and (3) macrophage responses, polarization, foreign-body reactions, vascularization, ECM remodeling, and tissue integration. Terms within each concept block were combined with OR, and concept blocks were combined with AND where appropriate.

Studies were prioritized when they directly evaluated polymer–ADM/dECM hybrids, assessed macrophage or foreign-body responses together with downstream remodeling, provided longitudinal macrophage data, or investigated mechanisms relevant to polymer persistence, ECM-derived immunoregulation, or temporally controlled macrophage responses. Studies limited to material fabrication or physicochemical characterization without relevant host-response data were excluded.

Hybrid studies were classified by their relevance to the proposed temporal framework. Tier 1 required comparable macrophage-state assessments at two or more post-implantation time points together with in vivo tissue-remodeling outcomes obtained in the same experimental model and implantation site. Tier 2 included macrophage-state and remodeling data but lacked either comparable longitudinal macrophage assessment or within-model/site linkage between the immune and remodeling outcomes. Tier 3 primarily supported tissue repair or material feasibility without adequately linking macrophage states to remodeling outcomes. Bulk-tissue cytokine or protein changes were not counted as longitudinal macrophage-state evidence unless attributable to macrophages.

Tier assignment was based on the availability and longitudinal coverage of macrophage-specific assessments and their relationship to tissue-remodeling outcomes. The tiers reflect mechanistic relevance to the proposed framework rather than overall methodological quality, risk of bias, or causal evidence strength. Tier 1 does not imply coverage of all phases from initiation through transition to late remodeling. Other features were recorded separately and did not determine tier: ECM source and material format, component controls (including polymer-only and ECM-only groups), macrophage sampling times, tissue outcomes, and applicability and attribution limits (Table 1). No formal risk-of-bias assessment was performed because the included studies were heterogeneous in design, model, and outcome measures and because this review is narrative rather than systematic. Study quality was therefore not graded. Nevertheless, potential confounding from incomplete component controls, additional bioactive agents, heterogeneous experimental models, and limited temporal sampling was considered when interpreting the evidence. Findings from broader polymer–ECM systems informed mechanistic extrapolation rather than direct validation of mADM-specific synergy.

## 3. Macrophage-Mediated Host Response Relevant to Polymer–mADM Hybrids

Although direct time-resolved evidence from polymer–mADM hybrids remains limited, their early tissue response can still be understood within the broadly conserved host-response cascade that follows biomaterial implantation. Immediately after injection, proteins from blood and interstitial fluid adsorb onto the material surface, forming a provisional biointerface [30]. mADM additionally retains structural and biochemical cues derived from its native ECM [1,30]. The composition and organization of this early interface are shaped by material properties such as surface chemistry, charge, wettability, roughness, porosity, and particle morphology. These features can alter protein adsorption and conformation and, in turn, affect complement activation, leukocyte adhesion, and early inflammatory signaling [31].

Injection-associated tissue injury and material recognition initiate an acute inflammatory response, with early neutrophil recruitment and activation of tissue-resident macrophages followed by the arrival of circulating monocytes. These monocytes differentiate into macrophages and accumulate at the material–tissue interface and within accessible ECM-rich regions of mADM [32]. Once recruited, macrophages respond to several local inputs, including adsorbed proteins, cytokines, material physicochemical properties, degradation products, and ECM-derived signals. Their functional states therefore continue to change as the surrounding microenvironment evolves [9,32].

The M1/M2 framework remains useful as functional shorthand, although biomaterial-associated macrophages occupy heterogeneous and transitional states [9,33]. Here, M1-like/pro-inflammatory and resolving/pro-remodeling describe functional tendencies rather than discrete phenotypes. Mixed states can influence fibroblast-mediated ECM organization [34], and M2-associated markers such as cluster of differentiation 206 (CD206), cluster of differentiation 163 (CD163), and arginase 1 (Arg1) may also occur in fibrotic remodeling. These markers should therefore be interpreted together with longitudinal evidence of inflammatory resolution and tissue organization [9,33,35].

Temporal evolution adds another dimension to macrophage behavior. A transient early M1-like/pro-inflammatory phase can support debris clearance and the recruitment of immune and stromal cells, whereas prolonged inflammatory activation may promote chronic foreign-body responses and fibrosis [9]. An experimental regenerative biomaterial study further showed that preserving an early M1-like/pro-inflammatory phase followed by a timely transition toward resolving/pro-remodeling macrophage states can improve regenerative outcomes, supporting polarization timing as an important regulatory variable [20].

When particles or aggregates cannot be completely engulfed, persistent adhesion and frustrated phagocytosis can drive macrophage fusion into multinucleated FBGCs [10]. Although FBGCs can contribute to material degradation, vascular changes, and tissue remodeling, persistent or excessive activity may accompany chronic inflammation, fibrotic encapsulation, or granulomatous reactions [10]. Macrophages also link inflammation to remodeling through reciprocal interactions with fibroblasts, endothelial cells, and the ECM, thereby regulating matrix synthesis, angiogenesis, and ECM turnover [18,35].

Macrophage activity and biomaterial degradation are also reciprocally coupled. Macrophages and FBGCs can contribute to material breakdown through degradative enzymes, reactive oxygen species, and local acidification, while the resulting degradation products can in turn influence macrophage activation [10,36]. The nature of this feedback appears to differ between synthetic polymers and ECM-derived materials. In biodegradable polyester systems such as PLGA, acidic degradation products can sustain M1-like/pro-inflammatory macrophage responses [36]. Degradation of mADM, by contrast, can release bioactive ECM-derived signals with different immunoregulatory effects through proteolytic remodeling [25,26]. These material-specific degradation–immune interactions are considered separately in the following sections.

**Figure 1:**
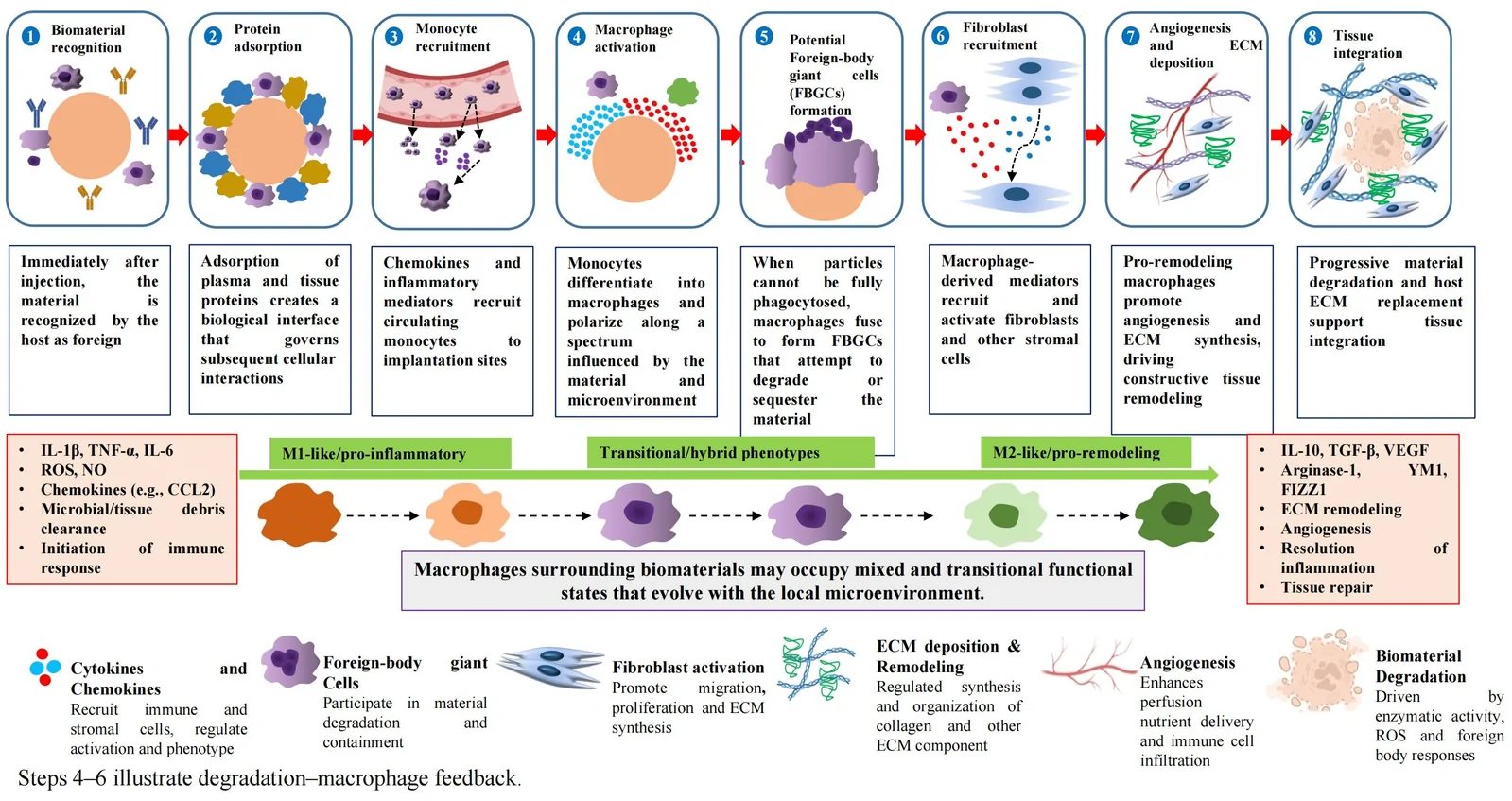
Macrophage-mediated host response and phenotypic dynamics during polymer–mADM hybrid remodeling. Created in BioRender. Pham, N. C. (2026) https://BioRender.com/pfyc0gv, accessed on 17 September 2026. BioRender publication license agreement no. NG2AB9G61L.

Within this context, the host response to polymer–mADM hybrids can be viewed as a dynamic and overlapping sequence: host–material interface formation → early inflammatory-cell recruitment → macrophage accumulation and activation → evolution of mixed or transitional macrophage states → macrophage–stromal cell interactions and degradation-associated feedback → angiogenesis and ECM remodeling → tissue integration. Frustrated phagocytosis and FBGC formation may also occur when the material cannot be efficiently engulfed [9,10,30,34] (Figure 1). We therefore consider the evolution of macrophage behavior over time, rather than a single phenotype measured at one time point, to provide a more useful biological basis for interpreting polymer–mADM remodeling [9,20].

## 4. Polymer-Driven Macrophage Activation as a Basis of Biostimulation

Although biodegradable synthetic polymers share a broadly similar foreign-body response framework, they do not elicit identical macrophage kinetics. The persistence of polymer materials within tissue largely determines how long macrophages remain exposed to the implant and its degradation products, thereby shaping the duration and temporal profile of the local inflammatory response [11,37].

We group these polymers by the material feature most relevant to the host response. PLLA/PDLLA illustrate particle persistence and inflammation-associated neocollagenesis [23,38], PLGA illustrates tunable degradation and acidic degradation products [36], and PCL/PLCL illustrate prolonged persistence and mechanical tunability [39,40].

### 4.1. PLLA and PDLLA: Particle Persistence and Inflammation-Associated Neocollagenesis

PLLA is one of the most established injectable biostimulatory polymers. Its clinical effect develops largely through the gradual host response to PLLA particles that persist within the tissue, rather than through long-term physical filling. Histological studies have shown macrophage and fibroblast accumulation around PLLA microspheres together with time-dependent collagen deposition. Type III collagen tends to predominate earlier, whereas type I collagen becomes more prominent at later stages [23]. PLLA biostimulation therefore follows a progressive course in which persistent particle stimulation is accompanied by cellular recruitment and subsequent neocollagenesis.

Macrophages act upstream of fibroblast activation in this process. PLLA can induce macrophage inflammatory signaling that contributes to the local microenvironment supporting subsequent stromal-cell recruitment and collagen synthesis [41]. Continued recognition of persistent particles may also prolong macrophage and FBGC activity. The biological effect of PLLA is therefore closely linked to how long the particles remain in tissue and how the host response evolves over that period [41,42].

PDLLA shares the same lactic-acid polymer backbone as PLLA but differs in stereochemistry and crystallinity. Our previous study showed that PLLA is relatively stereoregular and semicrystalline, whereas PDLLA is predominantly amorphous, resulting in differences in particle stability, degradation behavior, and macrophage-associated tissue responses [42]. Both materials are capable of stimulating neocollagenesis, although their inflammatory kinetics and the duration of macrophage exposure may differ [38,42].

### 4.2. PLGA: Degradation Kinetics and the Local Inflammatory Microenvironment

A distinctive feature of PLGA is that its degradation kinetics can be tuned by the lactide-to-glycolide ratio, molecular weight, and polymer structure [43,44]. PLGA generally undergoes bulk hydrolytic degradation, in which water penetrates the polymer matrix and progressive ester-bond cleavage reduces molecular weight before substantial mass loss occurs. As degradation proceeds, acidic oligomers and monomers can accumulate within the material, particularly when their generation exceeds local diffusion and clearance, and this local acidification can further influence subsequent degradation behavior [43,44]. Hydrolytic degradation ultimately produces lactic acid, glycolic acid, and intermediate oligomers, creating a chemical microenvironment that changes as degradation progresses [36,44].

Acidic PLGA degradation products can promote M1-like/pro-inflammatory macrophage activation [36]. In macrophages derived from the human THP-1 monocytic cell line, increasingly acidic extracts increased C-C chemokine receptor type 7 expression and the production of tumor necrosis factor-α (TNF-α) and interleukin-1β [36]. Macrophage responses therefore vary with degradation stage, local acidity, buffering, and clearance: rapid degradation may intensify short-term acidic exposure, whereas slower degradation prolongs cell–material interaction [36,43,44]. PLGA thus illustrates how degradation kinetics couple material persistence to changing macrophage stimuli.

### 4.3. PCL and PLCL: Prolonged Persistence and Host–Material Interactions

Compared with polylactic acid (PLA)-based polymers, PCL degrades more slowly and persists longer within tissue, making it suitable for applications that require sustained structural support [39]. Its prolonged persistence also extends the period of macrophage–material interaction. Experimental studies suggest that the host response to PCL is influenced by degradation behavior as well as by the ability of the material architecture to support cellular infiltration and tissue integration [45,46]. PLCL, a copolymer of L-lactide and ε-caprolactone, offers greater flexibility in tuning elasticity, mechanical compliance, and degradation behavior through changes in copolymer composition [40,47]. From a biomaterial-design perspective, its main advantage is the ability to provide a persistent and mechanically adaptable environment for long-term tissue remodeling. Direct evidence linking standalone injectable PLCL particles to macrophage-mediated neocollagenesis remains limited, and recent injectable studies have mainly incorporated PLCL microspheres into bioactive composite systems [48]. We therefore consider PLCL primarily as a tunable biodegradable platform in which material persistence and the mechanical microenvironment can be adjusted, while a specific macrophage polarization effect has yet to be established.

### 4.4. Context Dependence of Polymer-Induced Host Responses: Implications from Dermal Substitutes

The persistence and degradation of PLLA/PDLLA, PLGA, and PCL/PLCL can modulate macrophage responses, fibroblast activation, and matrix deposition, contributing to biostimulation [9,35]. However, sustained inflammation and FBGC activity may also accompany adverse fibrosis [9,10]. These outcomes depend on material properties and the local tissue environment, rather than polymer class alone.

Unlike the injectable applications discussed above, NovoSorb Biodegradable Temporising Matrix (BTM) and Integra Dermal Regeneration Template are dermal substitutes used for wound repair. BTM comprises a porous biodegradable polyurethane matrix and a temporary sealing layer, whereas Integra combines a cross-linked collagen–glycosaminoglycan matrix with a silicone covering [49]. A prospective clinical study reported BTM integration and improved scar quality during follow-up. In this cohort, the mean split-thickness skin graft take was 81.9%, and BTM integration remained possible in some wounds with signs suggestive of infection when appropriately managed [50]. Mouse studies comparing BTM with Integra showed host-cell infiltration and neovascularization in both templates. At two weeks, BTM exhibited greater inflammatory activity and more extensive vascularization, whereas Integra showed features of more advanced ECM remodeling [49,51]. These findings indicate that early inflammation can coexist with tissue integration and should not, by itself, be interpreted as evidence of inevitable adverse fibrosis. Although BTM may be associated with favorable tissue integration and scar outcomes, these observations alone do not establish an M2-like macrophage profile. Longitudinal macrophage characterization would be required to determine whether a resolving macrophage response contributes to these outcomes. Studies of collagen scaffolds further show that pore architecture, surface ligands, and degradation rate influence cell behavior and wound contraction [52]. Thus, polymer persistence or degradation should not be equated with adverse fibrosis without considering material composition, architecture, and tissue context.

The scaffold formats and wound environments of BTM and Integra differ from those of injectable polymer–mADM systems. These findings therefore provide contextual evidence rather than direct validation of polymer–mADM synergy. The proposed spatiotemporal overlap of material-derived signals and its immunological effects requires testing under defined formulation, architecture, degradation, and tissue conditions. mADM can supply tissue-derived ECM cues, but their effects depend on cue preservation, accessibility, and remodeling [53,54].

## 5. mADM as an Immunoregulatory ECM Microenvironment

### 5.1. Retained ECM Cues Create a Biologically Instructive Microenvironment

The retained collagen-rich architecture of mADM supports cell attachment and migration, while matrix-associated ligands and their spatial presentation regulate recognition through integrins and other matrix-sensing receptors [55,56,57,58]. Its fibrous and porous structure also facilitates cellular and vascular ingrowth, enabling progressive host-mediated remodeling [6,56].

### 5.2. ECM Degradation as an Immunoregulatory Signal

During remodeling, proteolysis can expose or release bioactive ECM sequences, including matricryptic peptides, that influence cell recruitment, macrophage behavior, angiogenesis, and matrix turnover [25,26]. Cellular infiltration and matrix breakdown therefore change the availability of ECM-derived signals over time, in contrast to synthetic polymer degradation, whose biological effects depend more directly on polymer chemistry and degradation products [36].

### 5.3. mADM Supports Macrophage-Directed Constructive Remodeling

Macrophages link mADM remodeling to stromal activity, angiogenesis, matrix turnover, and host ECM deposition [59,60,61,62]. Evidence from processed ECM materials suggests that the ECM environment can shape macrophage behavior during host remodeling, but it does not establish a fixed M1-to-M2 sequence or a temporal mechanism of synergy in polymer–mADM hybrids. We therefore treat mADM as an ECM environment that can modulate macrophage responses, rather than as an inherently “M2-inducing” material [54].

### 5.4. The Shared Biological Trade-Off of mADM: Biological Instruction Versus Structural Persistence

The principal advantage of mADM lies in its retention of tissue-derived ECM cues that are largely absent from synthetic polymers. These cues can support cellular infiltration and immunomodulation while also promoting vascularization and host tissue replacement [27]. Because these regenerative functions occur alongside enzymatic degradation and matrix remodeling, the structural persistence of mADM depends on how scaffold loss is matched by host tissue replacement [13]. As the implanted matrix is progressively remodeled, its initial mechanical contribution may decline unless newly formed host tissue provides sufficient replacement.

Processing strongly influences the biologically instructive capacity of mADM. Incomplete decellularization can leave cellular material, deoxyribonucleic acid (DNA), or antigens that sustain adverse responses, whereas overly aggressive processing can damage ECM proteins, deplete glycosaminoglycans, and disrupt structures needed for cell recognition and integration [57,63,64]. Effective processing must therefore remove potentially immunogenic material while preserving functional ECM architecture and signals. Source and lot also matter: donor species, anatomical site, donor age, and decellularization conditions can alter retained ECM composition, residual immunogenic material, and remodeling behavior [28,65]. Crosslinking, micronization, sterilization, and particle preparation further affect matrix structure, degradation, and cellular accessibility; Section 9.2 discusses their implications [12,65].

## 6. Proposed Temporal Macrophage-Switching Model for Polymer–mADM Synergy

Section 3, Section 4 and Section 5 provide three foundations for the proposed model. First, polymer persistence and degradation can influence host responses. Second, ECM degradation and remodeling can alter bioactive cues. Third, macrophage responses vary over time. We therefore hypothesize that polymer-associated stimulation and mADM-derived ECM cues overlap in space and time and change in relative contribution during implantation. We refer to this relationship as temporal coupling. Temporal coupling may jointly shape macrophage population states and tissue remodeling and thereby contribute to synergy.

Because these foundations arise mainly from separate material systems, they provide indirect support rather than direct validation of polymer–mADM synergy. Unless otherwise indicated, the statements below are hypotheses or predictions of the model; cited studies support individual biological components rather than the complete mechanism.

Within this model, macrophage switching refers to changes over time in the distribution of functional states within local macrophage populations, rather than necessarily to the conversion of individual cells. These populations may include pro-inflammatory, mixed or transitional, and resolving/pro-remodeling states [9,66]. Polymer and mADM are not assigned fixed M1- and M2-inducing roles, respectively, because both components may influence macrophages concurrently. Accordingly, changes in marker-positive cell proportions across time points do not, by themselves, demonstrate sequential state transitions within the same macrophages.

**Figure 2:**
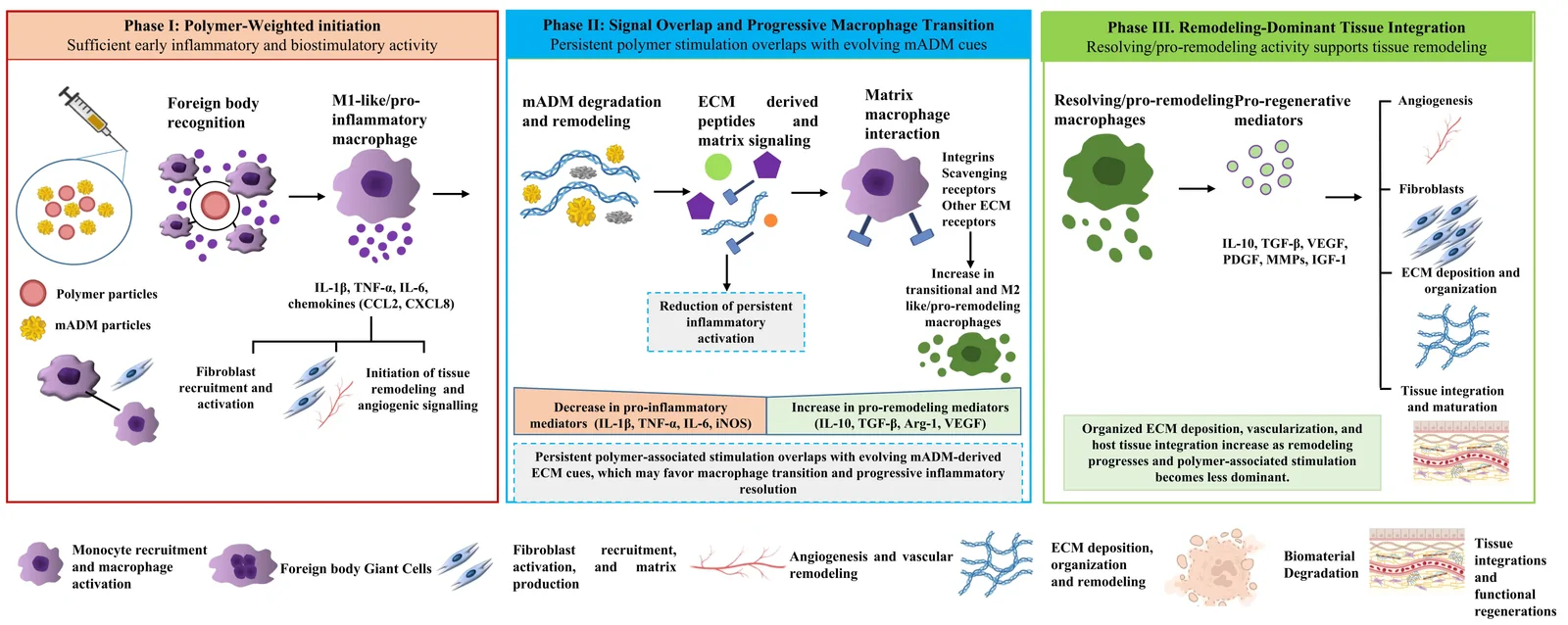
Temporal coupling of polymer-associated stimulation and evolving mADM-derived remodeling cues. Created in BioRender. Pham, N. C. (2026) https://BioRender.com/pfyc0gv, accessed on 17 September 2026. BioRender publication license agreement no. QO2AB9GD3C.

The model comprises three overlapping phases: (1) early material-associated initiation, (2) sustained temporal coupling with changes in macrophage population states, and (3) remodeling-dominant tissue integration (Figure 2). These phases describe a favorable trajectory to be tested, rather than a universal sequence or timetable. Section 6.1 and Section 6.2 explain the proposed trajectory, whereas Section 6.3 defines its testable predictions.

### 6.1. Early Material Cues and Initiation of Biostimulation

In this model, polymer-associated stimulation is assumed to make a larger relative contribution to the early host response when the polymer-derived stimulus is strong. This contribution depends on component ratio, dose, spatial distribution, and degradation/remodeling kinetics. mADM provides biochemical and structural ECM cues from the time of implantation, and their effects evolve with cellular infiltration, matrix degradation, and the accessibility of bioactive sites [25,26]. The model therefore allows both components to act from the outset, while their relative contributions shift over time rather than follow a fixed polymer-first sequence. A favorable early response is defined functionally as one that supports immune and stromal cell recruitment without progressing to a persistent adverse foreign-body response. Studies of temporally controlled immunomodulation in bone regeneration provide indirect support for preserving appropriate early inflammation to facilitate subsequent repair [20,21]. However, the required intensity and duration of this response, and the relative contribution of each material component, remain to be determined in polymer–mADM formulations.

### 6.2. Signal Overlap and State Changes in Macrophage Populations

During polymer persistence, mADM can undergo cellular infiltration and enzymatic remodeling, exposing macrophages to cues from both components within the same local microenvironment. The magnitude and spatial pattern of temporal coupling are expected to change with polymer degradation, tissue ingrowth, and matrix turnover. ECM studies show that ligand accessibility and spatial presentation, matrix–receptor interactions, and bioactive degradation fragments can regulate cell behavior [25,26,58]. These findings provide indirect mechanistic support but do not establish temporal coupling in polymer–mADM systems. Under favorable temporal coupling, the model predicts progressive enrichment of mixed, transitional, or resolving/pro-remodeling states within local macrophage populations, accompanied by vascular and matrix remodeling. This population-level trajectory remains to be tested. An increase in a single M2-associated marker is insufficient to demonstrate a beneficial state transition or an antifibrotic effect [9].

### 6.3. Testable Predictions for Temporal Coupling and Constructive Remodeling

Macrophage responses can interact bidirectionally with material degradation, vascularization, and ECM remodeling [9,26,34]. Temporal association between macrophage-state changes and remodeling therefore cannot, by itself, establish causal direction.

If temporal coupling contributes to synergy, altering component ratio, spatial distribution, or degradation/remodeling kinetics should modify temporal coupling and reproducibly change macrophage trajectories and tissue outcomes. Testing requires polymer-only, mADM-only, and hybrid groups, together with vehicle and additional-component controls where relevant. Sampling should cover initiation, transition, and late remodeling according to the kinetics of each system, with joint assessment of macrophage states, FBGC activity, component-specific degradation, vascularization, and ECM organization. Establishing causality further requires perturbing macrophage states or the timing of one material-derived input while controlling for other effects on cells and material properties.

The model would be weakened if, despite adequate sampling and assay sensitivity, remodeling improved without a distinguishable macrophage trajectory, or if state changes emerged only after the remodeling they are proposed to explain. Verified changes in the relative timing of the two material-derived inputs that leave macrophage trajectories and tissue outcomes unchanged would likewise argue against temporal coupling. Finally, superiority of the hybrid over either component alone does not establish synergy beyond additivity; this requires showing that the hybrid exceeds the outcome expected if the effects of the two components were merely additive. Section 7 evaluates the available evidence against these predictions.

## 7. Current Evidence for Polymer–ECM/Polymer–ADM Hybrid Systems

This section applies the tier criteria defined in Section 2 to assess support for the temporal mechanistic framework proposed in Section 6, focusing on changes in macrophage states over time and their links to tissue remodeling. ECM source, material format, component controls, and additional bioactive components are considered separately to distinguish observed findings, mechanistic extrapolations, and predictions requiring validation. Table 1 summarizes the 16 included studies. The tiers reflect the scope of support for the framework, not study quality or the strength of causal evidence.

### 7.1. Macrophage Responses in Polymer–ECM/Polymer–ADM Hybrid Systems

Available polymer–ECM hybrid studies generally indicate that ECM incorporation can alter macrophage responses within polymer-containing biomaterials, although relatively few studies have resolved these changes longitudinally.

Among the Tier 1 studies, a PLCL–adipose dECM/collagen scaffold showed a higher CD206/CD68 staining-area ratio than PLCL alone at both 4 and 8 weeks [67]. A PCL–placental ECM vascular graft provided a broader time course, with CD68, TNF-α, inducible nitric oxide synthase (iNOS), and CD206 profiles changing across 1, 2, 4, and 12 weeks and early differences becoming less pronounced at later stages [68]. A polymer-layered pericardial dECM composite was assessed for iNOS- and CD163-associated macrophage responses at 1, 4, and 12 weeks; both signals remained detectable at 12 weeks, indicating persistence of a mixed macrophage-associated profile at the population level [69]. The small intestinal submucosa (SIS)-decorated scaffold study assessed CD206 at days 3 and 7, but its later bone-repair outcomes were obtained from a different implantation site. It was therefore classified as Tier 2 despite providing longitudinal immune observations [70]. These findings suggest that hybrid-associated macrophage responses are time-dependent and cannot be adequately characterized by a single M1- or M2-associated endpoint.

An injectable PLCL porous microsphere–muscle dECM system provides a particularly informative comparison. In the formulations without added interleukin-4 (IL-4) or insulin-like growth factor-1 (IGF-1), dECM, PLCL microspheres, and their hybrid were evaluated at 1 and 4 weeks. The hybrid showed no consistent advantage in iNOS or CD206 expression but, at 4 weeks, had fewer FBGCs and greater cellular infiltration into the porous microspheres than the microsphere-only group [48]. This pattern suggests that the overall course of the foreign-body response and tissue integration may be more informative than directional changes in individual polarization markers. A PCL/ADM–PCL/decellularized adipose tissue (DAT) bilayer skin construct provides Tier 2 evidence, with CD86/CD163 assessment at 1 month and accompanying tissue-remodeling outcomes [71]. This construct lacked an ADM-only control and included an additional DAT layer, limiting attribution to ADM; direct polymer–mADM temporal coupling remains untested.

Tier 2 studies extend this association to other tissue settings. Mesenchymal stem cell (MSC)-derived ECM reduced the foreign-body response to PLGA and increased M2-associated macrophage accumulation [72]; PCL–muscle dECM scaffolds increased Arg1-positive macrophage activity together with myofiber regeneration [73]. More recent PCL–vascular dECM and PCL–cell-derived ECM systems likewise associated shifts toward M2-associated/resolving macrophage profiles with vascular or tissue regeneration [74,75].

### 7.2. Vascularization, ECM Remodeling, and Tissue Integration Following Hybridization

For macrophage modulation to be relevant to constructive regeneration, it should be accompanied by corresponding tissue-level changes, including vascularized tissue ingrowth, organized matrix remodeling, and progressive host–material integration.

This pattern is evident across the principal Tier 1 studies. PLCL–adipose dECM constructs showed greater vascular maturation and adipose-like tissue formation than PLCL alone [67]. PCL–placental ECM grafts also linked longitudinal changes in macrophage profiles with enhanced endothelialization, smooth-muscle maturation, and ECM deposition [68]. In the injectable PLCL–muscle dECM system, host cells, capillaries, and newly deposited matrix progressively entered the porous microsphere network [48]. A Tier 2 ADM-containing dermal–adipose construct also showed enhanced vascularization and more organized collagen remodeling during dermal–adipose reconstruction [71].

Related hybrid systems provide additional evidence for this downstream association. Pericardial dECM–polymer composites supported vascular-cell infiltration and local matrix remodeling [69]. PCL-based vascular dECM constructs supported endothelialization and reduced neointimal hyperplasia, whereas PLCL-based constructs promoted smooth-muscle regeneration and ECM deposition [74,76]. A recent cell-derived ECM/fibrin/PCL scaffold was also associated with reduced inflammatory macrophage activity, increased vascular perfusion, and more organized tissue repair [75].

Studies with limited temporal macrophage data support individual downstream components, although they do not resolve the proposed macrophage trajectory. PLGA nanoparticles loaded with interleukin-8 (IL-8) incorporated into ADM together with transplanted MSCs enhanced capillary formation and collagen deposition [77], while a PCL–ADM nerve conduit supported axonal regeneration and functional recovery [78]. The thymosin β4 (Tβ4)-loaded dECM–PLGA system provided macrophage-state data at day 14 alongside tissue repair outcomes, supporting Tier 2 classification. In this case, sustained delivery of thymosin β4 makes it difficult to attribute these effects specifically to the polymer–ECM interaction [79]. A sodium tanshinone IIA sulfonate (STS)-loaded PCL–meniscus ECM scaffold also showed early M2-associated macrophage polarization and subsequent meniscal repair, although STS loading and cell seeding limit attribution to the polymer–ECM combination [80].

Hybrid outcomes also depend on the therapeutic purpose. A thermosensitive formulation containing micronized human ADM and synthetic polymers reduced postoperative adhesions. At day 7, cell counts for the macrophage-associated markers ED1 and F4/80 showed different patterns: ED1-positive cells decreased, whereas F4/80-positive cells did not differ significantly among groups [81]. This example shows that the biological effect of combining polymer and ADM depends strongly on formulation and tissue context, together with the intended therapeutic purpose.

### 7.3. Study Controls and Temporal Limitations of Current Evidence

Control groups help distinguish the contributions of individual material components. The basic comparison in the PLCL microsphere–muscle dECM study included microsphere-only, ECM-only, and hybrid groups and should be distinguished from the IL-4/IGF-1-loaded repair formulations [48]. The PLCL–adipose dECM system also contained type I collagen, whereas the PCL–placental ECM system involved heparinization and IL-4 loading. Neither study included the corresponding ECM hydrogel or matrix alone as a control [67,68]. These control limitations and additional components should be recorded separately: they limit attribution to the polymer–ECM combination itself but should not independently determine tier assignment.

Temporal coverage was limited in most studies. Five of the 16 studies assessed macrophage states at two or more in vivo time points [48,67,68,69,70], only two at three or more [68,69], and four measured immune and remodeling outcomes within the same model and implantation site [48,67,68,69]. The remaining studies used one principal macrophage time point, lacked macrophage-state assessment, or provided insufficient timing information; most therefore could not demonstrate sequential switching. A late increase in CD206 does not establish sequential switching, and two time points may miss initiation or transition. Changes in population composition also do not prove switching within the same cells. Serial bulk-tissue measurements in Hao et al. lacked macrophage-specific attribution [71], whereas the SIS study assessed immunity and bone repair at different sites [70]. Macrophage state and remodeling times should therefore be reported separately with the model and implantation site.

Tier 1 therefore indicates longitudinal in vivo macrophage-state observations alongside remodeling outcomes but does not establish the proposed temporal mechanism. Tier 2 may still include pathway-specific mechanistic evidence, as illustrated by transforming growth factor-β-induced protein (TGFβI) perturbation in the MSC-derived ECM study [72], but such evidence does not by itself validate temporal coupling in polymer–mADM systems. Table 1 summarizes tier assignments, component controls, sampling times, and interpretive limitations.

**Table 1:** Mechanistic summary of polymer–ECM/ADM hybrid studies relevant to the proposed temporal macrophage-switching framework.

Study and Tier	Hybrid System and Material Relevance	Component Controls and Co-Interventions	In Vivo Macrophage and Foreign-Body-Response Evidence	Tissue-Remodeling Evidence	Primary Interpretive Limit
Li et al. [48] Tier 1—same-site longitudinal	Other ECM; injectable. Porous PLCL microspheres combined with a muscle-derived ECM (mECM) hydrogel.	P ✓/E ✓/H ✓; IL-4/IGF-1-loaded arms analyzed separately.	Weeks 1 and 4: iNOS, CD206, and FBGCs; no consistent iNOS/CD206 advantage, but fewer FBGCs and greater pore infiltration at week 4.	Cellular infiltration, capillary ingrowth, and new matrix/collagen deposition were assessed at 1 and 4 weeks.	Two time points; muscle-derived ECM; IL-4/IGF-1-loaded results require separate interpretation.
Lee et al. [67] Tier 1—same-site longitudinal	Other ECM; non-injectable scaffold. 3D-printed PLCL combined with adipose dECM/type I collagen hydrogel.	P ✓/E ✗/H ✓; type I collagen was an additional matrix component.	Weeks 4 and 8: higher CD206/CD68-positive area ratio in the hybrid than in PLCL alone.	Vascular maturation and adipose-like tissue formation were evaluated at 4 and 8 weeks.	Early response not sampled; added collagen and absent dECM-only control limit attribution.
Liu et al. [68] Tier 1—same-site longitudinal	Other ECM; vascular scaffold. PCL microfibers combined with placental ECM nanofibers, heparinized with or without IL-4 loading.	P ✓/E ✗/H ✓; heparin and IL-4 were additional interventions.	Weeks 1, 2, 4, and 12: CD68, TNF-α, iNOS, and CD206; early group differences diminished by week 12.	Endothelialization, vascular smooth-muscle maturation, and ECM deposition were followed through 12 weeks.	Non-dermal ECM; heparin/IL-4 and absent ECM-only control limit attribution.
Mudigonda et al. [69] Tier 1—same-site longitudinal	Other ECM; cardiovascular patch. PCL/chitosan nanofiber layer combined with decellularized bovine pericardium.	P ✗/E ✓/H ✓; fixed and decellularized pericardium served as ECM comparators.	Weeks 1, 4, and 12: iNOS and CD163; both signals remained detectable at week 12.	Host-cell infiltration, glycosaminoglycan deposition, collagen preservation, and local matrix remodeling were assessed across implantation times.	Non-dermal patch; no polymer-only control; mixed markers do not establish sequential switching.
Tan et al. [70] Tier 2—no within-site linkage	Other ECM; bone scaffold. SIS-coated 3D-printed PLGA/HAp/icariin construct.	P ✓/E ✗/H ✓; HAp and icariin were additional components.	Days 3 and 7: CD206-related responses in a subcutaneous model; bone repair assessed separately.	Angiogenesis, osteogenesis, and bone regeneration were evaluated at 4 and 8 weeks in a diabetic bone-defect model.	Different immune and repair sites; CD206 only; co-components and absent SIS-only control limit attribution.
Hao et al. [71] Tier 2—no longitudinal macrophage assessment	ADM-related; non-injectable skin construct. PCL/ADM dermal layer combined with a PCL/DAT adipose layer.	P ✓/E ✗/H ✓; DAT was an additional ECM component.	Month 1: CD86/CD163; bulk-tissue protein data at months 1 and 3 lacked macrophage specificity.	Wound closure, vascularization, collagen organization, and adipose regeneration were assessed at 1 and 3 months.	No longitudinal macrophage-specific assessment; added DAT and absent ADM-only control limit attribution.
Deng et al. [72] Tier 2—no longitudinal macrophage assessment	Other ECM; cell-derived matrix. PLGA scaffold modified with human umbilical-cord MSC-derived ECM.	P ✓/E ✗/H ✓; TGFβI knockdown provided pathway perturbation.	Day 3: increased CD206^+^/Ly6C^−^ macrophages; TGFβI knockdown attenuated the response.	Foreign-body response and osteogenic tissue formation were evaluated in vivo.	Single in vivo immune time point; pathway evidence does not establish temporal coupling.
Patel et al. [73] Tier 2—limited temporal coverage	Other ECM; muscle scaffold. Aligned PCL nanofibers incorporating skeletal muscle dECM.	P ✓/E ✗/H ✓; an untreated group was also included.	Day 28: increased arginase-positive macrophages.	Myofiber regeneration was examined at days 7 and 28; muscle mass and force were not restored.	Late phenotype association; non-dermal ECM and no dECM-only comparator.
Xu et al. [74] Tier 2—limited temporal coverage	Other ECM; vascular graft. Heparin-coated bilayer PCL/decellularized vascular ECM construct.	P ✓/E NR/H ✓; heparinization was an additional modification.	M2-associated neointimal accumulation reported; immune sampling time NR.	The graft supported endothelialization and reduced neointimal smooth-muscle proliferation/hyperplasia at 2 months.	Temporal coverage and ECM-only control NR; heparin and multistep fabrication limit attribution.
He et al. [75] Tier 2—single immune endpoint	Other ECM; cell-derived matrix scaffold. MSC-derived decellularized matrix in a fibrin/PCL construct.	P ✓/E ✗/H ✓; fibrin was an additional component.	Week 2: lower CD86 and higher CD206 than fibrin/PCL.	Tissue integration, bone repair, and vascular perfusion were assessed at later endpoints.	Single in vivo phenotype endpoint; no matrix-only implant; fibrin was an additional component.
Liang et al. [79] Tier 2—single immune endpoint	Other ECM; injectable uterine formulation. Decidualized endometrial ECM hydrogel combined with Tβ4-loaded PLGA microspheres.	P ✓/E ✓/H ✓; Tβ4-loaded PLGA was an active intervention.	Day 14: CD86, iNOS, Arg1, and CD206 assessed by staining and F4/80-gated flow cytometry.	Fibrosis, angiogenesis, epithelial repair, and fertility recovery were evaluated.	Single immune time point; Tβ4 and non-dermal ECM limit binary material attribution.
Li M et al. [80] Tier 2—no within-site linkage	Other ECM; meniscal construct. STS-loaded PCL–meniscus ECM hydrogel hybrid, with a cell-seeded repair arm.	P NR/E NR/H ✓; STS and cell seeding were additional interventions.	Early subcutaneous macrophage modulation; marker panel and sampling time NR.	Meniscal regeneration and chondroprotection were evaluated in a rabbit defect model.	STS and cell seeding limit attribution; immune and repair outcomes came from different sites.
Shi et al. [76] Tier 3—no macrophage–remodeling linkage	Other ECM; vascular graft. Tri-layered PLCL construct containing layer-specific arterial dECM and salidroside.	P ✓/E ✗/H ✓; salidroside was an active intervention.	No macrophage-state profiling reported; vascular remodeling assessed.	Endothelialization, smooth-muscle regeneration, ECM deposition, and antithrombotic performance were assessed.	Salidroside co-intervention; no macrophage-state linkage to vascular remodeling.
Zhang et al. [77] Tier 3—macrophage evidence NR	ADM-related; cell-delivery construct. PLGA@IL-8 nanoparticles loaded into ADM for MSC delivery.	P NR/E NR/H ✓; IL-8 and MSCs were major co-interventions.	Macrophage-state/foreign-body-response assessment NR.	Wound closure, capillary formation, collagen deposition, and MSC retention were assessed.	IL-8 and MSC co-interventions; component controls and temporal immune evidence NR.
Kim et al. [78] Tier 3—no linked macrophage-state evidence	ADM-related; non-injectable nerve conduit. 3D-printed PCL combined with porcine ADM.	P ✗/E ✗/H ✓; only an unmatched conduit comparator was included.	No macrophage phenotyping reported.	Axonal regeneration and functional recovery were assessed at 4 and 12 weeks.	No matched PCL-only or ADM-only controls; non-injectable nerve conduit.
Jeong et al. [81] Tier 3—cell counts only	ADM-related; injectable anti-adhesion formulation. Thermosensitive polymer/HA combined with micronized human ADM.	P ✗/E ✗/H ✓; only unmatched commercial anti-adhesion comparators were included.	Day 7: ED1 counts decreased, F4/80 was unchanged, and polarization was not assessed.	Postoperative adhesion, microvessel and collagen density, and material absorption were evaluated.	Unmatched commercial controls; anti-adhesion endpoint; no macrophage-state trajectory.

Note: Tier 1 includes comparable in vivo macrophage-state assessments at two or more post-implantation time points plus remodeling outcomes in the same model and implantation site. Tier 2 includes macrophage-state and remodeling data but lacks either longitudinal macrophage assessment or within-model/site linkage. Tier 3 supports repair or material feasibility without adequate macrophage–remodeling linkage. P, target-polymer comparator without ECM/ADM; E, relevant ECM/ADM comparator without polymer; H, polymer–ECM/ADM hybrid; ✓, included in the corresponding in vivo comparison; ✗, confirmed absent; NR, not reported or insufficiently described. Labels indicate group presence, not full matching. Tiers indicate relevance to the proposed framework, not study quality or causality. “ADM-related” denotes direct ECM-source relevance; “other ECM” denotes mechanistic extrapolation. ADM, acellular dermal matrix; DAT, decellularized adipose tissue; dECM, decellularized extracellular matrix; ECM, extracellular matrix; FBGC, foreign-body giant cell; HAp, hydroxyapatite; HA, hyaluronic acid; mECM, muscle-derived extracellular matrix; MSC, mesenchymal stem cell; PCL, polycaprolactone; PLCL, poly(L-lactide-co-ε-caprolactone); PLGA, poly(lactic-co-glycolic acid); SIS, small intestinal submucosa; Tβ4, thymosin β4.ED1 and F4/80, macrophage-associated markers; Ly6C, lymphocyte antigen 6 complex, locus C.

### 7.4. Evidence Synthesis and Implications for the Macrophage Polarization Window

Available studies report differences in macrophage and foreign-body responses alongside vascularization, cellular infiltration, and ECM remodeling, although temporal patterns and tissue outcomes vary across material systems [48,67,68,69]. These findings support a dynamic view of host–material interactions but do not uniformly demonstrate a sequential transition from pro-inflammatory to repair-associated macrophage states.

The evidence assessed here does not establish causal links among the spatiotemporal overlap of material cues, macrophage population trajectories, and potential polymer–mADM synergy. Hybrid superiority over a single component, or macrophage-state changes coinciding with tissue improvement, does not demonstrate mediation by temporal coupling. Testing this mechanism requires matched component controls, sampling across key phases, and the targeted perturbations outlined in Section 6.3.

Direct material evidence comes from ADM/mADM-containing bilayer skin scaffolds, PLGA@IL-8/ADM constructs for MSC delivery, PCL–ADM nerve conduits, and an injectable thermosensitive anti-adhesion formulation [71,77,78,81]. These studies are directly relevant to the target ECM source but do not test temporal coupling in a standardized injectable polymer–mADM formulation. Indirect evidence comes from systems incorporating muscle-, adipose-, placental-, pericardial-, vascular-, endometrial-, meniscal-, small-intestinal-submucosa-, or cell-derived ECM [48,67,68,69,70,72,73,74,75,76,79,80]. These studies support mechanistic extrapolation rather than direct inference for polymer–mADM systems because the ECM source, material format, tissue setting, or additional bioactive components differ.

The proposed macrophage polarization window should therefore be treated as a functionally defined interval requiring validation in specific formulations and tissue environments, rather than an established universal switching time. Its assessment should integrate inflammatory resolution, foreign-body responses, vascularization, and ECM organization, rather than rely on an isolated increase in an M2-associated marker.

## 8. Biological Interpretation of the Macrophage Polarization Window

**Figure 3:**
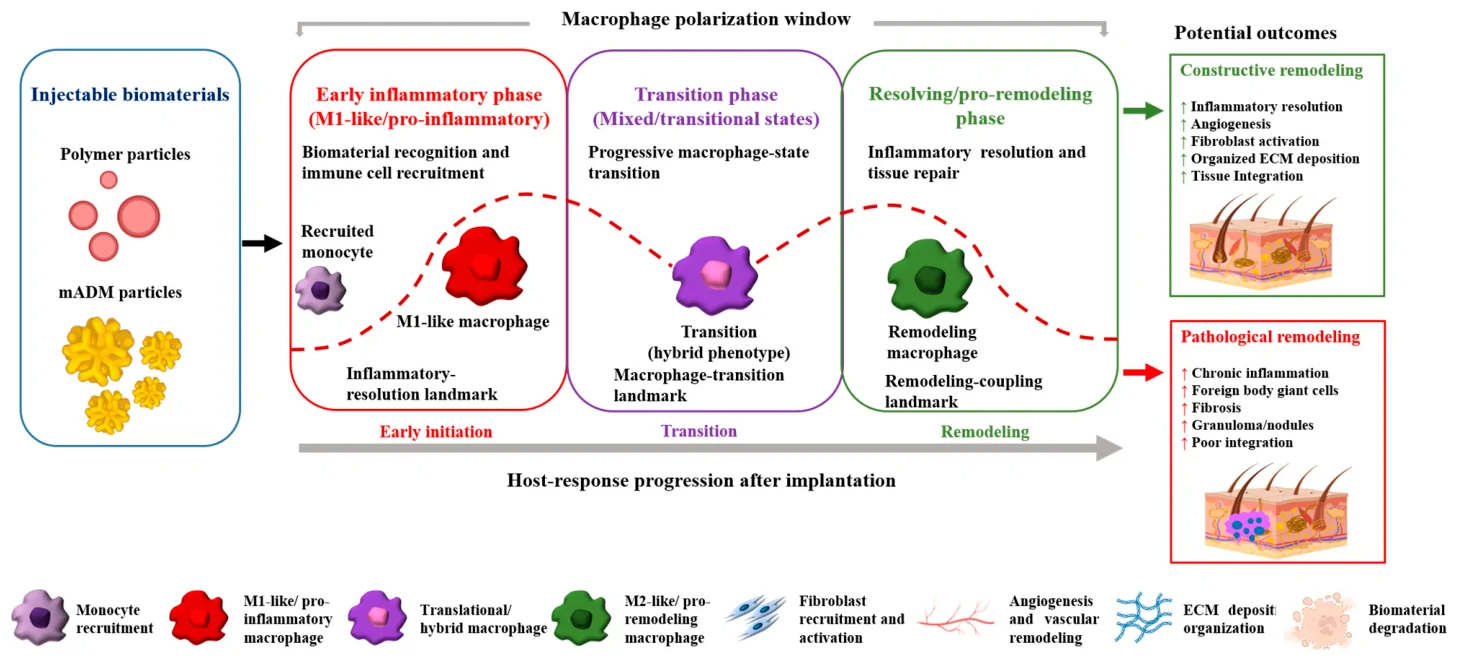
Biological landmarks of the macrophage polarization window in injectable biomaterial remodeling. Created in BioRender. Pham, N. C. (2026) https://BioRender.com/pfyc0gv, accessed on 17 September 2026. BioRender publication license agreement no. NU2AB9GKMS. Red, purple, and green denote early inflammatory, transitional, and resolving/pro-remodeling states, respectively. Green and red outcome boxes indicate constructive and pathological remodeling, respectively. The gray horizontal arrow indicates host-response progression after implantation. Arrows indicate the proposed progression or potential outcomes. The dashed curve is schematic and does not represent quantitative measurements. Cell and material symbols are defined in the legend.

We use the term macrophage polarization window to describe the favorable functional trajectory within the temporal macrophage-switching model. This window is a biologically defined interval, not a fixed chronological one. It begins when early inflammatory initiation is sufficient to support cell recruitment and ends when the response consolidates into constructive integration or deviates toward persistent foreign-body remodeling. Section 8.1 interprets phase 1 of the model. The inflammatory-resolution and macrophage-transition landmarks in Section 8.2 interpret phase 2, whereas the remodeling-coupling landmark bridges phase 2 to phase 3. Section 8.3 describes deviation from the favorable trajectory rather than a fourth phase. The landmarks proposed here (Figure 3) are interpretive criteria and have not been validated in polymer–mADM systems.

### 8.1. Sufficient Early Inflammatory Initiation

In this model, polymer-associated stimulation is assumed to make a larger relative contribution to early host activation when the polymer-derived stimulus is strong, while mADM-derived cues are present from implantation (Section 6.1). The adequacy of this initiating response is judged by functional events, including recruitment of immune and stromal cells, engagement of fibroblasts and endothelial cells, and the early appearance of angiogenic and matrix-remodeling activity. The model predicts that suppressing the response before these events are established could weaken subsequent recruitment, biostimulation, and remodeling. Sufficient early initiation is therefore better viewed as a functional threshold for regenerative activation than as a fixed post-implantation time point.

### 8.2. Biological Landmarks of the Transition Phase

After sufficient early initiation, the model predicts a gradual transition in macrophage population states. This phase is evaluated through three proposed operational landmarks rather than a single M1-to-M2 switch at one time point. Their interpretation requires longitudinal response patterns, not fixed marker thresholds or predetermined chronological cutoffs.

Inflammatory-resolution landmark: An initially activated inflammatory program begins to stabilize or decline across serial assessments. Evaluation should integrate inflammation-associated readouts such as TNF-α, iNOS, and CD86 with the persistence of macrophage and FBGC activity [9,10]. Persistent or increasing activity would indicate failure to establish resolution; the direction of change after initial activation is therefore more informative than the absolute value of any single marker. A hybrid vascular-graft study illustrates how macrophage-associated differences can change across implantation times [68], although it does not establish a universal threshold.

Macrophage-transition landmark: Transition requires a longitudinal change in the functional composition of the macrophage population, with declining inflammation-associated activity accompanied by an increasing contribution of mixed, transitional, and resolving/pro-remodeling states. Because macrophages with combined M1/M2 characteristics can regulate fibroblast-mediated ECM assembly and architecture [34], a CD86-to-CD206 crossover alone provides limited evidence of transition. A stronger assessment combines multiple markers and, where feasible, multiparametric phenotyping to demonstrate a population-level shift in functional state.

Remodeling-coupling landmark: Macrophage transition becomes biologically meaningful when its temporal evolution is linked to constructive tissue remodeling. The model predicts that a favorable macrophage shift should coincide with or precede increasing cellular infiltration and vascular ingrowth, followed by ECM turnover and organized host-matrix deposition. Tier 1 studies report associations between macrophage-related measures and these outcomes [48,67,68] but do not establish their sequence or causal relationship (Section 7.3). Reduced persistence of FBGC-dominant remodeling may provide additional supporting evidence [48]. Stronger support would require longitudinal tracking of immune and tissue events within the same experimental model and implantation site.

### 8.3. Biological Signs of Deviation Toward Persistent Foreign-Body Remodeling

Deviation from the favorable trajectory is suggested when inflammatory activity fails to resolve and macrophage/FBGC accumulation persists alongside profibrotic macrophage–fibroblast interactions, progressive fibrotic organization, or restricted cellular and vascular infiltration [10,35]. These features should be interpreted collectively rather than from any single marker.

This progression is unlikely to follow a universal day- or week-based timeline. Indirect evidence from a magnetically actuated scaffold system showed that the timing of the same macrophage-modulating mechanical stimulus altered macrophage phenotype and subsequent vascular/foreign-body responses, whereas delayed intervention produced substantially weaker effects [22]. Host-response plasticity may therefore depend on the implantation stage. Loss of a favorable trajectory is more clearly indicated by persistent macrophage/FBGC activity together with progressive fibrotic organization and impaired tissue integration.

The macrophage polarization window thus provides a functional interpretation of the temporal macrophage-switching model described in Section 6. Because immune kinetics differ across biomaterial systems, shared biological landmarks are more informative for cross-system comparison than a universal switching time. In polymer–mADM hybrids, the trajectory is expected to depend on the magnitude and persistence of polymer-associated stimulation, the evolving accessibility of mADM-derived cues, and their temporal coupling; the relevant material determinants are discussed in Section 9.

## 9. Determinants of Macrophage Switching in Polymer–mADM Hybrid Injectable Biomaterials

**Figure 4:**
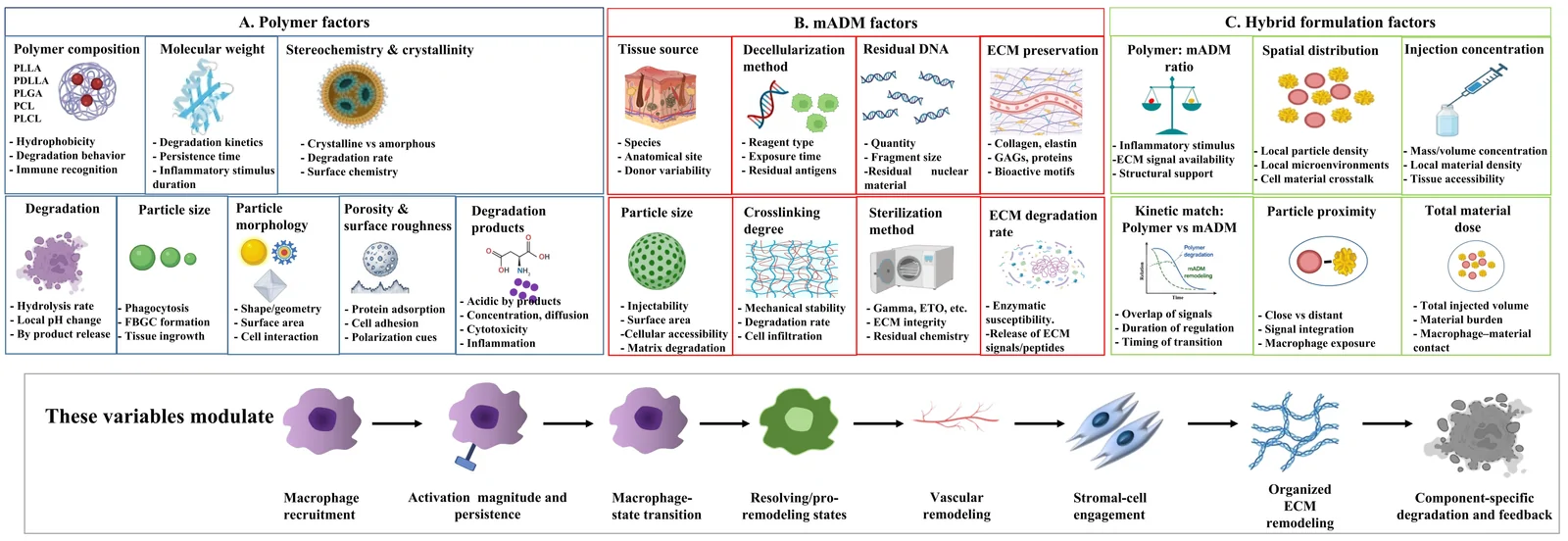
Material and formulation determinants of the macrophage response trajectory in polymer–mADM hybrid injectable biomaterials. Created in BioRender. Pham, N. C. (2026) https://BioRender.com/pfyc0gv, accessed on 17 September 2026. BioRender publication license agreement no. BJ2AB9GQE9. Blue, red, and green outlines distinguish polymer factors, mADM factors, and hybrid formulation factors, respectively. Illustrations represent the material properties and biological processes identified by their accompanying labels. Arrows in the lower panel schematically link macrophage responses with tissue remodeling and component-specific degradation; they do not imply a fixed sequence or established causal relationships.

In polymer–mADM hybrids, the macrophage trajectory and biological landmarks described in Section 6, Section 7 and Section 8 depend on properties at both the component and formulation levels. Polymer composition and particle architecture shape the magnitude and persistence of material-associated stimulation. mADM source, processing, particle properties, and lot variability shape the availability and evolution of ECM-derived cues. Component ratio, spatial organization, kinetics, and total dose determine their temporal coupling. Figure 4 summarizes these determinants.

### 9.1. Polymer Composition, Particle Architecture, and Early Stimulation

Polymer composition, molecular weight, hydrophobicity, and crystallinity shape degradation kinetics and tissue persistence, which in turn influence the duration of macrophage-associated stimulation. These properties primarily regulate the intensity and history of material exposure rather than imposing a fixed macrophage phenotype. PLA stereochemistry, for example, can produce distinct degradation-linked immunometabolic and cytokine responses [82]. From a design perspective, sufficient material persistence is needed to support early regenerative initiation, while excessive or prolonged stimulation may favor persistent foreign-body activation.

Quantitative degradation data should support this relationship. In a recent porous PCL filler, the weight-average molecular-weight half-life was approximately 52 weeks at 37 °C; microspheres remained detectable in vivo at 18 months, whereas more than 90% had disappeared by 24 months [83]. Although system-specific, these values illustrate how material persistence can be quantified and related to the proposed macrophage trajectory. Degradation also changes the local chemical environment: rapid breakdown may increase short-term exposure to degradation products, whereas slower degradation prolongs macrophage–material interaction [84].

Particle architecture further shapes the macrophage–material interface. In PLLA/PDLLA implants, material morphology was a stronger determinant of inflammation and FBGC formation than surface chemistry [37], while powdery and smooth PCL microstructures produced different local immune environments [46]. In one PLGA system, 5.8-μm microspheres were phagocytosed, whereas 29.8-μm monodisperse and 34.1-μm polydisperse particles were not. Giant-cell formation around the larger particles was only occasional [85]. When complete engulfment is prevented, persistent cell–material contact may produce incomplete or frustrated phagocytic engagement and, depending on material properties and local signals, favor macrophage fusion into FBGCs [10,85]. This boundary is not defined by diameter alone, because particle morphology, three-dimensional geometry, porosity, and surface texture also influence macrophage–material interactions and tissue ingrowth [37,46,86]. A porous injectable PCL filler used 25–50-μm microspheres [83], but this formulation-specific range should not be considered optimal for other polymers or mADM; it provides a mechanistic hypothesis rather than an mADM-specific design rule.

### 9.2. mADM Source, Processing, Particle Properties, and Batch Variability

Residual DNA remains a widely used quality-control metric. Commonly cited decellularization criteria include <50 ng double-stranded DNA/mg dry ECM, DNA fragments <200 bp, and the absence of visible nuclear material [87]. More recent human-tissue studies continue to use ≤50 ng/mg dry weight as a practical reference value [88]. These criteria are useful as quality benchmarks, although they do not directly predict macrophage behavior.

Crosslinking and related processing modify matrix persistence, cellular infiltration, and vascularization [89,90]. Excessive or insufficient stabilization may delay remodeling or shorten scaffold persistence. Oxygen-controlled γ-irradiation at 25–30 kGy with annealing can reduce radiation-associated damage while preserving collagen integrity [91].

For injectable mADM, micronization can alter injectability, surface area, cellular accessibility, and enzymatic remodeling. A recent microparticulate human ADM retained ECM components and growth factors and supported fibroblast infiltration and neovascularization with a reduced capsular response [92], although it did not establish a particle-size–response relationship. Reported mADM preparations vary widely: particulate ADM studies evaluated size fractions ranging from 194–380 μm to 856–1485 μm, a wound-applied human mADM paste had a mean particle size of 664.2 ± 389.9 μm, and a recent ultra-fine injectable human ADM reported a mean size of approximately 70 μm [93,94,95]. These formulations differed in processing, delivery route, and tissue model, and none isolated particle size while tracking longitudinal macrophage or FBGC responses. They therefore establish formulation-specific feasibility rather than an optimal mADM size or phagocytic threshold. Future studies should report the full particle-size distribution together with shape, surface texture, porosity, injectability, and degradation.

Together, source- and process-dependent differences create batch-to-batch variability in collagen architecture, glycosaminoglycan retention, residual immunogenic material, enzymatic susceptibility, cue accessibility, and remodeling kinetics [28,65,96,97,98]. Nominally equivalent mADM lots may therefore generate different macrophage trajectories within the same polymer formulation and limit comparisons across studies using different ECM sources and processing methods. Future studies should evaluate multiple independent lots and predefine lot-release criteria for tissue-source qualification, particle-size distribution, residual DNA, retained ECM composition, degradation, and injectability.

### 9.3. Hybrid Formulation Determinants of Spatial and Temporal Coupling

The polymer:mADM ratio is one important determinant of the relative contribution of each component. Increasing the polymer fraction may increase local material burden and prolong polymer-associated stimulation. A higher mADM fraction may increase the availability of tissue-derived ECM cues while also changing the structural and degradation characteristics of the composite. The biological effect of this ratio is unlikely to follow a simple linear pattern and needs to be interpreted in the context of particle architecture, total material dose, and the surrounding tissue environment. In an injectable composite consisting of microspheres and short microfibers, variation in component ratio significantly altered macrophage recruitment and polarization, with higher microfiber fractions associated with greater scar formation [86]. A more directly relevant PLCL–muscle dECM system evaluated muscle dECM:microsphere mass ratios of 30:1, 10:1, 5:1, and 3:1 and selected a 10:1 ratio with a 3% muscle dECM hydrogel for subsequent studies [48]. These studies show that component ratio can be systematically adjusted, although they do not identify a universal polymer:ECM ratio for hybrid systems.

Spatial organization is another important variable because it determines whether macrophages encounter polymer-associated and ECM-derived signals within the same local microenvironment. Spatial confinement and pore architecture can directly influence macrophage morphology and inflammatory signaling while also affecting tissue infiltration [99]. In the PLCL–muscle dECM injectable system, porous microspheres formed an interpenetrating structure with the muscle-derived ECM (mECM) hydrogel, and ECM was distributed both within and around the microsphere network [48]. This type of spatial integration may increase the likelihood that cells interacting with persistent polymer surfaces are also exposed to progressively remodeled ECM. Future polymer–mADM studies should therefore characterize overall component ratio together with local particle density, interparticle spacing, polymer–mADM contact, pore accessibility, and the distribution of mADM around polymer particles.

Kinetic matching directly connects hybrid formulation to the temporal model proposed in Section 6: the design objective is sufficient temporal overlap between persistent polymer-associated stimulation and evolving mADM-derived cues. Spatiotemporally programmable biomaterials are increasingly being explored as a way to influence dynamic macrophage-state transitions [29]. In the PLCL–mECM system, the mECM hydrogel was largely degraded by week 4, whereas PLCL microspheres remained present and continued to support cellular infiltration and matrix deposition [48]. This example demonstrates component-specific kinetics without defining an optimal temporal relationship. Future studies would be more informative if polymer degradation and mADM remodeling were quantified in parallel and related to longitudinal changes in macrophage states, FBGC formation, vascularization, and host ECM deposition.

Injection concentration and total material dose add another level of control and should be considered separately from component ratio. Two formulations with the same polymer:mADM ratio may generate different biological responses when the total amount of implanted material differs. A higher material burden can prolong macrophage–material contact and restrict tissue accessibility, potentially favoring a more persistent or disorganized inflammatory environment [100]. Hybrid studies should therefore report component ratio alongside mass or volume concentration, total injected volume, local material density, and component-specific distribution. These variables should be evaluated against longitudinal macrophage and remodeling outcomes rather than treated only as manufacturing specifications.

## 10. Clinical Translation of Injectable Polymer–mADM Hybrids

### 10.1. Current Clinical Landscape and Translational Status

Synthetic biodegradable polymer injectables already have an established clinical base. PLLA-based Sculptra, PDLLA-based AestheFill, and PCL-based Ellansé-M have been clinically evaluated for facial soft-tissue correction [101,102,103].

Clinical evidence for injectable human ADM is also emerging. In 2026, a randomized, double-blind, split-face trial showed that injectable particulated human acellular dermal matrix improved skin density and elasticity over 20 weeks [104]. A separate multicenter, double-blind randomized non-inferiority trial found that mADM achieved correction of moderate-to-severe nasolabial folds comparable to that of a cross-linked collagen filler through 6 months [105].

To our knowledge, no commercially established injectable co-formulation combining mADM with PLLA, PDLLA, PLGA, PCL, or PLCL has been reported. A 2026 retrospective study of 206 patients found improved outcomes after combined PLLA and ADM injections for abdominal skin laxity and striae gravidarum [106], but it evaluated combined administration rather than a standardized co-formulated product. The principal translational gap is therefore the development of a reproducible injectable hybrid with product-level manufacturing controls and regulatory validation.

### 10.2. From Experimental Hybrid Systems to Standardized Clinical Products

Clinical translation requires the material-design variables discussed in Section 9 to be converted into reproducible final-product specifications. Relevant attributes extend beyond the polymer:mADM ratio and particle composition. Suspension homogeneity after reconstitution, sedimentation or aggregation, injection force, needle compatibility, and component uniformity per delivered volume all need to be controlled. Reconstitution conditions can substantially affect particle dispersion in injectable polylactic-acid formulations, highlighting the need for formulation-specific preparation standards [107].

Within the proposed framework, these specifications are biological test variables as well as product-control parameters. Polymer:mADM ratio, total dose, and component distribution may determine the relative strength and spatial overlap of polymer-associated and mADM-derived cues. Degradation/remodeling kinetics may determine their temporal overlap, whereas injectability and lot consistency may affect local dispersion, cue accessibility, and the reproducibility of macrophage trajectories and remodeling outcomes (Section 9.2 and Section 9.3). Product development should therefore pair physicochemical release criteria with longitudinal assessments of macrophage states, FBGC activity, and tissue remodeling. These relationships remain hypotheses until tested in standardized polymer–mADM co-formulations.

As discussed in Section 9.2, mADM source and processing can generate lot-to-lot differences in ECM composition, degradation behavior, and cue accessibility [28,65,96,97,98]. Translation therefore requires predefined acceptance criteria for source-tissue qualification, decellularization, micronization, component mixing, and the final co-formulation.

Sterilization and storage must also be validated for the final co-formulation. Sterilization can alter the physicochemical properties and biological activity of dECM [108], and conditions compatible with synthetic polymers may compromise mADM integrity. The selected process must therefore maintain sterility while preserving polymer stability, ECM integrity, injectability, and component distribution throughout shelf life.

Regulatory classification and evidence requirements will depend on product composition, tissue source, processing, and intended use [98,109]. Safety and performance should therefore be evaluated using the final co-formulation rather than inferred from either component alone. Long-term preclinical studies should jointly track macrophage-state trajectories, persistent inflammation and FBGC activity, nodules or granulomas, fibrosis, component degradation, and tissue integration.

## 11. Limitations and Future Validation

Direct evidence remains limited because standardized injectable polymer–mADM co-formulations have rarely been studied; most evidence comes from polymer–ECM systems differing in ECM source, anatomy, and material format. Existing studies do not link material-cue overlap to longitudinal macrophage trajectories and remodeling, and most sample macrophages at only one or two time points (Section 7.3). Macrophage state is usually inferred from a few M1/M2-associated markers, so temporal association cannot establish causality. The effects of mADM lot variability, particle size, and morphology also remain untested directly in polymer–mADM systems (Section 9.1 and Section 9.2). Finally, this narrative review provides no quantitative synthesis or formal risk-of-bias assessment; Tier assignments indicate relevance to the framework rather than study quality or causal strength (Section 2).

Future work should compare polymer-alone, mADM-alone, and hybrid formulations under standardized conditions and across several independent mADM lots. Sampling should be adapted to material kinetics and span early initiation, transition, and late remodeling, with joint assessment of macrophage states, FBGC activity, component-specific degradation, vascularization, and ECM organization. Deliberately varying the polymer:mADM ratio and component-specific kinetics, and where feasible manipulating macrophage-state transitions, would show whether these variables predictably change the proposed macrophage-to-remodeling sequence.

## 12. Conclusions

We propose that potential polymer–mADM synergy may arise from temporal coordination between persistent polymer-associated stimulation and remodeling-dependent mADM cues. This model predicts a trajectory from early inflammatory activation through transitional states to resolving/pro-remodeling activity, defined by functional landmarks rather than fixed times. Existing polymer–ECM studies are compatible with the model but remain largely indirect, temporally sparse, and noncausal. Validation requires standardized polymer-only, mADM-only, and hybrid comparisons across several mADM lots, with longitudinal immune and remodeling assessments and deliberate variation in component ratio, dose, and kinetics. If supported, these variables could inform design and release specifications for more predictable injectable products.

## Data Availability

No new data were created or analyzed in this study. Data sharing is not applicable to this article.

## Supplementary Materials

The following supporting information can be downloaded at: https://www.mdpi.com/article/10.3390/ijms27209239/s1.

## Abbreviations

The following abbreviations are used in this manuscript:

ADM	Acellular dermal matrix
CD68	Cluster of differentiation 68
CD86	Cluster of differentiation 86
CD163	Cluster of differentiation 163
CD206	Cluster of differentiation 206
Arg1	Arginase 1
BTM	Biodegradable Temporising Matrix
DAT	Decellularized adipose tissue
dECM	Decellularized extracellular matrix
DNA	Deoxyribonucleic acid
ECM	Extracellular matrix
FBGC	Foreign-body giant cell
IGF-1	Insulin-like growth factor-1
IL-4	Interleukin-4
IL-8	Interleukin-8
iNOS	Inducible nitric oxide synthase
mADM	Micronized acellular dermal matrix
mECM	Muscle-derived extracellular matrix
MSC	Mesenchymal stem cell
PCL	Polycaprolactone
PDLLA	Poly-D,L-lactic acid
PLA	Polylactic acid
PLCL	Poly(L-lactide-co-ε-caprolactone)
PLGA	Poly(lactic-co-glycolic acid)
PLLA	Poly-L-lactic acid
SIS	Small intestinal submucosa
STS	Sodium tanshinone IIA sulfonate
TGFβI	Transforming growth factor-β-induced protein
TNF-α	Tumor necrosis factor-α
Tβ4	Thymosin β4
ED1	Rat CD68-associated macrophage marker
F4/80	Macrophage-associated surface marker
Ly6C	Lymphocyte antigen 6 complex, locus C

## References

[1] Ostadi Y., Khanali J., Tehrani F.A., Yazdanpanah G., Bahrami S., Niazi F., Niknejad H. (2024). Decellularized Extracellular Matrix Scaffolds for Soft Tissue Augmentation: From Host-Scaffold Interactions to Bottlenecks in Clinical Translation. Biomater. Res..

[2] Cho K.H., Uthaman S., Park I.K., Cho C.S. (2018). Injectable Biomaterials in Plastic and Reconstructive Surgery: A Review of the Current Status. Tissue Eng. Regen. Med..

[3] Xu P., Kankala R.K., Wang S., Chen A. (2024). Decellularized extracellular matrix-based composite scaffolds for tissue engineering and regenerative medicine. Regen. Biomater..

[4] Stein P., Vitavska O., Kind P., Hoppe W., Wieczorek H., Schürer N.Y. (2015). The biological basis for poly-L-lactic acid-induced augmentation. J. Dermatol. Sci..

[5] Haddad S., Galadari H., Patil A., Goldust M., Al Salam S., Guida S. (2022). Evaluation of the biostimulatory effects and the level of neocollagenesis of dermal fillers: A review. Int. J. Dermatol..

[6] Chang L.S., Kim S.H., Kim H., Ryu S., Choy Y.B., Kim S.W. (2023). Evaluation of Paste-Type Micronized Acellular Dermal Matrix for Soft Tissue Augmentation: Volumetric and Histological Assessment in a Mouse Model. Aesthetic Plast. Surg..

[7] Ko H., Kim D., Shin C., Gong N.Y., You B., Oh H.S., Lee J., Oh S.H. (2023). In Vivo Efficacy of an Injectable Human Acellular Dermal Matrix. Aesthetic Plast. Surg..

[8] Chu C., Liu L., Rung S., Wang Y., Ma Y., Hu C., Zhao X., Man Y., Qu Y. (2020). Modulation of foreign body reaction and macrophage phenotypes concerning microenvironment. J. BioMed Mater. Res. A.

[9] Martin K.E., García A.J. (2021). Macrophage phenotypes in tissue repair and the foreign body response: Implications for biomaterial-based regenerative medicine strategies. Acta Biomater..

[10] Cai F., Jiang B., He F. (2024). Formation and biological activities of foreign body giant cells in response to biomaterials. Acta Biomater..

[11] Kyriakides T.R., Kim H.J., Zheng C., Harkins L., Tao W., Deschenes E. (2022). Foreign body response to synthetic polymer biomaterials and the role of adaptive immunity. BioMed Mater..

[12] Qiao S., Peijie T., Nan J. (2024). Crosslinking strategies of decellularized extracellular matrix in tissue regeneration. J. BioMed Mater. Res. A.

[13] Stec E., Lombardi J., Augustin J., Sandor M. (2023). Acellular Dermal Matrix Susceptibility to Collagen Digestion: Effect on Mechanics and Host Response. Tissue Eng. Part A.

[14] Li S., Poche J.N., Liu Y., Scherr T., McCann J., Forghani A., Smoak M., Muir M., Berntsen L., Chen C. (2018). Hybrid Synthetic-Biological Hydrogel System for Adipose Tissue Regeneration. Macromol. Biosci..

[15] Xie X., Wang W., Cheng J., Liang H., Lin Z., Zhang T., Lu Y., Li Q. (2020). Bilayer pifithrin-α loaded extracellular matrix/PLGA scaffolds for enhanced vascularized bone formation. Colloids Surf. B Biointerfaces.

[16] Sheikh Z., Brooks P.J., Barzilay O., Fine N., Glogauer M. (2015). Macrophages, Foreign Body Giant Cells and Their Response to Implantable Biomaterials. Materials.

[17] Zhu F., Wang S., Zhu X., Pang C., Cui P., Yang F., Li R., Zhan Q., Xin H. (2023). Potential effects of biomaterials on macrophage function and their signalling pathways. Biomater. Sci..

[18] Wang Y., Fan Y., Liu H. (2021). Macrophage Polarization in Response to Biomaterials for Vascularization. Ann. BioMed Eng..

[19] Liu Y., Segura T. (2020). Biomaterials-Mediated Regulation of Macrophage Cell Fate. Front. Bioeng. Biotechnol..

[20] Huang D., Xu K., Huang X., Lin N., Ye Y., Lin S., Zhang J., Shao J., Chen S., Shi M. (2022). Remotely Temporal Scheduled Macrophage Phenotypic Transition Enables Optimized Immunomodulatory Bone Regeneration. Small.

[21] Qiao W., Xie H., Fang J., Shen J., Li W., Shen D., Wu J., Wu S., Liu X., Zheng Y. (2021). Sequential activation of heterogeneous macrophage phenotypes is essential for biomaterials-induced bone regeneration. Biomaterials.

[22] Steele L.A., Spiller K.L., Cohen S., Rom S., Polyak B. (2022). Temporal Control over Macrophage Phenotype and the Host Response via Magnetically Actuated Scaffolds. ACS Biomater. Sci. Eng..

[23] Yu H., Zhang Y.L., Chen Y., Dong Y.X., Hong W.J., Luo S.K. (2025). In Vivo Effectiveness of Poly-L-Lactic Acid Microsphere Dermal Fillers in Stimulating Collagen Synthesis. Aesthetic Plast. Surg..

[24] Ngan Giang N., Le L.T.T., Ngoc Chien P., Trinh T.T., Thi Nga P., Zhang X.R., Jin Y.X., Zhou S.Y., Han J., Nam S.Y. (2024). Assessment of inflammatory suppression and fibroblast infiltration in tissue remodelling by supercritical CO(2) acellular dermal matrix (scADM) utilizing Sprague Dawley models. Front. Bioeng. Biotechnol..

[25] He C., Yang Z., Jin Y., Qi X., Chu J., Deng X. (2018). ADM Scaffolds Generate a Pro-regenerative Microenvironment During Full-Thickness Cutaneous Wound Healing Through M2 Macrophage Polarization via Lamtor1. Front. Physiol..

[26] de Castro Brás L.E., Frangogiannis N.G. (2020). Extracellular matrix-derived peptides in tissue remodeling and fibrosis. Matrix Biol..

[27] Long J., Qin Z., Chen G., Song B., Zhang Z. (2023). Decellularized extracellular matrix (d-ECM): The key role of the inflammatory process in pre-regeneration after implantation. Biomater. Sci..

[28] Golebiowska A.A., Intravaia J.T., Sathe V.M., Kumbar S.G., Nukavarapu S.P. (2024). Decellularized extracellular matrix biomaterials for regenerative therapies: Advances, challenges and clinical prospects. Bioact. Mater..

[29] Li M., Feng Z., Zeng S., Jin M., Chen S., Yang H., Huang Y. (2026). Co-evolving biomaterials: Spatiotemporally programmable materials steering dynamic macrophage state transitions. Acta Biomater..

[30] Anderson J.M., Rodriguez A., Chang D.T. (2008). Foreign body reaction to biomaterials. Semin. Immunol..

[31] Chen Y., Luo Z., Meng W., Liu K., Chen Q., Cai Y., Ding Z., Huang C., Zhou Z., Jiang M. (2024). Decoding the “Fingerprint” of Implant Materials: Insights into the Foreign Body Reaction. Small.

[32] Piatnitskaia S., Rafikova G., Bilyalov A., Chugunov S., Akhatov I., Pavlov V., Kzhyshkowska J. (2024). Modelling of macrophage responses to biomaterials in vitro: State-of-the-art and the need for the improvement. Front. Immunol..

[33] Murray P.J., Allen J.E., Biswas S.K., Fisher E.A., Gilroy D.W., Goerdt S., Gordon S., Hamilton J.A., Ivashkiv L.B., Lawrence T. (2014). Macrophage activation and polarization: Nomenclature and experimental guidelines. Immunity.

[34] Witherel C.E., Sao K., Brisson B.K., Han B., Volk S.W., Petrie R.J., Han L., Spiller K.L. (2021). Regulation of extracellular matrix assembly and structure by hybrid M1/M2 macrophages. Biomaterials.

[35] Sudarsanam P.K., Alsema E.C., Beijer N.R.M., Kooten T.V., Boer J. (2024). Beyond Encapsulation: Exploring Macrophage-Fibroblast Cross Talk in Implant-Induced Fibrosis. Tissue Eng. Part B Rev..

[36] Ma S., Feng X., Liu F., Wang B., Zhang H., Niu X. (2021). The pro-inflammatory response of macrophages regulated by acid degradation products of poly(lactide-co-glycolide) nanoparticles. Eng. Life Sci..

[37] Lucke S., Walschus U., Hoene A., Schnabelrauch M., Nebe J.B., Finke B., Schlosser M. (2018). The in vivo inflammatory and foreign body giant cell response against different poly(l-lactide-co-d/l-lactide) implants is primarily determined by material morphology rather than surface chemistry. J. BioMed Mater. Res. A.

[38] Oh S., Seo S.B., Kim G., Batsukh S., Park C.H., Son K.H., Byun K. (2023). Poly-D,L-Lactic Acid Filler Increases Extracellular Matrix by Modulating Macrophages and Adipose-Derived Stem Cells in Aged Animal Skin. Antioxidants.

[39] Malikmammadov E., Tanir T.E., Kiziltay A., Hasirci V., Hasirci N. (2018). PCL and PCL-based materials in biomedical applications. J. Biomater. Sci. Polym. Ed..

[40] Zhang M., Chang Z., Wang X., Li Q. (2021). Synthesis of Poly(l-lactide-co-ε-caprolactone) Copolymer: Structure, Toughness, and Elasticity. Polymers.

[41] Nowag B., Schäfer D., Hengl T., Corduff N., Goldie K. (2024). Biostimulating fillers and induction of inflammatory pathways: A preclinical investigation of macrophage response to calcium hydroxylapatite and poly-L lactic acid. J. Cosmet. Dermatol..

[42] Jang J., Jin C., Ding Z., Kwak S., Lee H.S., Park Y., Chien P.N., Choi K., Heo C. (2026). PLLA and PDLLA Fillers: Linking Crystallinity and Mechanical Stability to Aggregation, Redispersion, and Collagen Formation. In Vivo.

[43] Walker J., Albert J., Liang D., Sun J., Schutzman R., Kumar R., White C., Beck-Broichsitter M., Schwendeman S.P. (2023). In vitro degradation and erosion behavior of commercial PLGAs used for controlled drug delivery. Drug Deliv. Transl. Res..

[44] Xu Y., Kim C.S., Saylor D.M., Koo D. (2017). Polymer degradation and drug delivery in PLGA-based drug-polymer applications: A review of experiments and theories. J. BioMed Mater. Res. B Appl. Biomater..

[45] Oh H., Lee S., Na J., Kim B.J., Kim J.H. (2022). Comparative Evaluation of Physical Characteristics and Preclinical Data of a Novel Monodisperse Polycaprolactone Microspheres Filler. Aesthetic Plast. Surg..

[46] Hou L., Meng Z., Zhang J., Jiao Y., Chang K., He J., Zhang J. (2025). Effect of 3D-Printed Polycaprolactone Scaffold With Powdery/Smooth Micromorphology on Local Immune Environments. J. BioMed Mater. Res. A.

[47] Bachtiar E.O., Ritter V.C., Gall K. (2021). Structure-property relationships in 3D-printed poly(l-lactide-co-ε-caprolactone) degradable polymer. J. Mech. Behav. BioMed Mater..

[48] Li Y., Liu S., Zhang J., Wang Y., Lu H., Zhang Y., Song G., Niu F., Shen Y., Midgley A.C. (2024). Elastic porous microspheres/extracellular matrix hydrogel injectable composites releasing dual bio-factors enable tissue regeneration. Nat. Commun..

[49] Banakh I., Cheshire P., Rahman M., Carmichael I., Jagadeesan P., Cameron N.R., Cleland H., Akbarzadeh S. (2020). A Comparative Study of Engineered Dermal Templates for Skin Wound Repair in a Mouse Model. Int. J. Mol. Sci..

[50] Lo C.H., Brown J.N., Dantzer E.J.G., Maitz P.K.M., Vandervord J.G., Wagstaff M.J.D., Barker T.M., Cleland H. (2022). Wound healing and dermal regeneration in severe burn patients treated with NovoSorb^®^ Biodegradable Temporising Matrix: A prospective clinical study. Burns.

[51] Cheshire P.A., Herson M.R., Cleland H., Akbarzadeh S. (2016). Artificial dermal templates: A comparative study of NovoSorb™ Biodegradable Temporising Matrix (BTM) and Integra(^®^) Dermal Regeneration Template (DRT). Burns.

[52] Yannas I.V., Tzeranis D., So P.T. (2015). Surface biology of collagen scaffold explains blocking of wound contraction and regeneration of skin and peripheral nerves. BioMed Mater..

[53] Rowley A.T., Nagalla R.R., Wang S.W., Liu W.F. (2019). Extracellular Matrix-Based Strategies for Immunomodulatory Biomaterials Engineering. Adv. Healthc. Mater..

[54] Xiao W., Yang Y., Chu C., Rung S.A., Wang Z., Man Y., Lin J., Qu Y. (2023). Macrophage response mediated by extracellular matrix: Recent progress. BioMed Mater..

[55] Brown M., Li J., Moraes C., Tabrizian M., Li-Jessen N.Y.K. (2022). Decellularized extracellular matrix: New promising and challenging biomaterials for regenerative medicine. Biomaterials.

[56] Dasgupta A., Orgill D., Galiano R.D., Zelen C.M., Huang Y.C., Chnari E., Li W.W. (2016). A Novel Reticular Dermal Graft Leverages Architectural and Biological Properties to Support Wound Repair. Plast. Reconstr. Surg. Glob. Open.

[57] Jin C., Zhang X., Jin Y., Chien P.N., Heo C.Y. (2025). Acellular Extracellular Matrix Scaffolds in Regenerative Medicine: Advances in Decellularization and Clinical Applications. J. Funct. Biomater..

[58] Kanchanawong P., Calderwood D.A. (2023). Organization, dynamics and mechanoregulation of integrin-mediated cell-ECM adhesions. Nat. Rev. Mol. Cell Biol..

[59] Liu X., Guo T., Huang Z., Chen S., Chen L., Li C., Tian T., Qian Y., Yang L., Xiang J. (2024). Acellular dermal matrix hydrogels promote healing of radiation-induced skin injury in a rat model. J. Mater. Chem. B.

[60] Savitri C., Ha S.S., Kwon J.W., Kim S.H., Kim Y.M., Park H.M., Kwon H., Ji M.J., Park K. (2024). Human Fibroblast-Derived Matrix Hydrogel Accelerates Regenerative Wound Remodeling Through the Interactions with Macrophages. Adv. Sci. (Weinh).

[61] Cottler P.S., Sun N., Thuman J.M., Bielak K.M.H., Salopek L.S., Piñeros-Fernandez A., Hu S., Campbell C.A. (2020). The Biointegration of a Porcine Acellular Dermal Matrix in a Novel Radiated Breast Reconstruction Model. Ann. Plast. Surg..

[62] Yu Y., Xiao H., Tang G., Wang H., Shen J., Sun Y., Wang S., Kong W., Chai Y., Liu X. (2023). Biomimetic hydrogel derived from decellularized dermal matrix facilitates skin wounds healing. Mater. Today Bio.

[63] Kasravi M., Ahmadi A., Babajani A., Mazloomnejad R., Hatamnejad M.R., Shariatzadeh S., Bahrami S., Niknejad H. (2023). Immunogenicity of decellularized extracellular matrix scaffolds: A bottleneck in tissue engineering and regenerative medicine. Biomater. Res..

[64] Mbele M., Jay T., Khumalo N.P., Bayat A. (2026). Low-detergent sonication decellularization preserves extracellular matrix architecture and enhances cell infiltration in human acellular dermal matrix. Front. Bioeng. Biotechnol..

[65] Mansour R.N., Karimizade A., Enderami S.E., Abasi M., Talebpour Amiri F., Jafarirad A., Mellati A. (2023). The effect of source animal age, decellularization protocol, and sterilization method on bovine acellular dermal matrix as a scaffold for wound healing and skin regeneration. Artif. Organs.

[66] Liang N.E., Parker J.B., Lu J.M., Januszyk M., Wan D.C., Griffin M., Longaker M.T. (2024). Understanding the Foreign Body Response via Single-Cell Meta-Analysis. Biology.

[67] Lee S., Lee H.S., Chung J.J., Kim S.H., Park J.W., Lee K., Jung Y. (2021). Enhanced Regeneration of Vascularized Adipose Tissue with Dual 3D-Printed Elastic Polymer/dECM Hydrogel Complex. Int. J. Mol. Sci..

[68] Liu S., Yao L., Wang Y., Li Y., Jia Y., Yang Y., Li N., Hu Y., Kong D., Dong X. (2023). Immunomodulatory hybrid micro-nanofiber scaffolds enhance vascular regeneration. Bioact. Mater..

[69] Mudigonda J., Onohara D., Amedi A., Suresh K.S., Kono T., Corporan D., Padala M. (2022). In vivo efficacy of a polymer layered decellularized matrix composite as a cell honing cardiovascular tissue substitute. Mater. Today Bio.

[70] Tan J., Chen Z., Xu Z., Huang Y., Qin L., Long Y., Wu J., Yang H., Chen X., Yi W. (2024). Small intestine submucosa decorated 3D printed scaffold accelerated diabetic bone regeneration by ameliorating the microenvironment. J. Mater. Chem. B.

[71] Hao Z., Guo Y., Jie J., Jiang W., Wang Z., Sun J., Guo N., Zhou M. (2026). Conjugate electrospinning dermal-adipose bilayered skin: Structural integration promoting regenerative skin remodeling. J. Nanobiotechnol..

[72] Deng M., Tan J., Hu C., Hou T., Peng W., Liu J., Yu B., Dai Q., Zhou J., Yang Y. (2020). Modification of PLGA Scaffold by MSC-Derived Extracellular Matrix Combats Macrophage Inflammation to Initiate Bone Regeneration via TGF-β-Induced Protein. Adv. Healthc. Mater..

[73] Patel K.H., Talovic M., Dunn A.J., Patel A., Vendrell S., Schwartz M., Garg K. (2020). Aligned nanofibers of decellularized muscle extracellular matrix for volumetric muscle loss. J. BioMed Mater. Res. B Appl. Biomater..

[74] Xu Z., Geng X., Ye L., Li L., Xing Y., Cheng J., Wang C., Gu Y., Tong Z., Guo L. (2025). Application of subcutaneous extracellular matrix to prepare bilayer heparin-coated polycaprolactone/decellularized small-diameter vascular graft for tissue regeneration. Int. J. Biol. Macromol..

[75] He Y., Hua C., Liang L., Huang C., Li Y., Huang J., Luo C., Zhang Z.Y. (2026). A Hybrid Mesenchymal-Stem-Cell-Derived Decellularized Matrix Scaffold Supports Bone Repair and Vascular Perfusion in Steroid-Associated Osteonecrosis. Biomater. Res..

[76] Shi J., Teng Y., Li D., He J., Midgley A.C., Guo X., Wang X., Yang X., Wang S., Feng Y. (2023). Biomimetic tri-layered small-diameter vascular grafts with decellularized extracellular matrix promoting vascular regeneration and inhibiting thrombosis with the salidroside. Mater. Today Bio.

[77] Zhang Y., Jiang W., Kong L., Fu J., Zhang Q., Liu H. (2023). PLGA@IL-8 nanoparticles-loaded acellular dermal matrix as a delivery system for exogenous MSCs in diabetic wound healing. Int. J. Biol. Macromol..

[78] Kim H.B., Kwon S., Kim Y.H., Eom J.S., Shim J.H., Han H.H. (2025). The Fabrication of a 3D-Printed Nerve Guidance Conduit Using Heterogeneous Composite Materials and Its Effectiveness on Sciatic Nerve Defects of a Rabbit Model. Polymers.

[79] Liang Y., Yu Z., Du S., Guo Y., Li J., Yan Y., Jin S., Liang W., Li M., Jin N. (2026). Decidualization-empowered ECM hydrogel integrating sustained Tβ4 release drives endometrial regeneration in intrauterine adhesions. Nat. Commun..

[80] Li M., Yin H., Chen M., Deng H., Tian G., Guo W., Yi G., Guo Q., Chen Z., Liu S. (2023). STS loaded PCL-MECM based hydrogel hybrid scaffolds promote meniscal regeneration via modulating macrophage phenotype polarization. Biomater. Sci..

[81] Jeong J.J., Lee D.W., Song S.Y., Park Y., Kim J.H., Kim J.I., Kim H.G., Nam K.T., Lee W.J., Nam K.H. (2019). Development of novel biocompatible thermosensitive anti-adhesive agents using human-derived acellular dermal matrix. PLoS ONE.

[82] Maduka C.V., Alhaj M., Ural E., Kuhnert M.M., Habeeb O.M., Schilmiller A.L., Hankenson K.D., Goodman S.B., Narayan R., Contag C.H. (2023). Stereochemistry Determines Immune Cellular Responses to Polylactide Implants. ACS Biomater. Sci. Eng..

[83] Kim J.S., Sung J.H., Kwon D.Y., Park J.E., Cho H., Yoon H.S. (2025). Biodegradability and Efficacy of Porous Polycaprolactone Microsphere Dermal Filler for Fine Lines. J. Cosmet. Dermatol..

[84] Umemori K., Little D. (2025). Impact of Polymer Degradation on Cellular Behavior in Tissue Engineering. Bioprinting.

[85] Zandstra J., Hiemstra C., Petersen A.H., Zuidema J., van Beuge M.M., Rodriguez S., Lathuile A.A., Veldhuis G.J., Steendam R., Bank R.A. (2014). Microsphere size influences the foreign body reaction. Eur. Cell Mater..

[86] Si L., Liu S., Gao C., Wang Z., Xiong F. (2026). Spatiotemporal Synergistic Effects of Injectable Biomaterials With Two Typical 3D Geometries on Macrophage Modulation for Enhanced Tissue Regeneration. Adv. Healthc. Mater..

[87] Crapo P.M., Gilbert T.W., Badylak S.F. (2011). An overview of tissue and whole organ decellularization processes. Biomaterials.

[88] Mineta S., Endo S., Ueno T. (2023). Optimization of decellularization methods using human small intestinal submucosa for scaffold generation in regenerative medicine. Int. J. Exp. Pathol..

[89] Chen Y., Zhang Y., Wang Q., Dan N., Li Y., Li Z., Dan W., Wang Y. (2023). Converting Acellular Dermal Matrix into On-Demand Versatile Skin Scaffolds by a Balanceable Crosslinking Approach for Integrated Infected Wounds Therapy. Biomacromolecules.

[90] Stefanelli V., Lombardi J., Ferrer J., Gardocki-Sandor M. (2025). Vascularization of Human Acellular Dermal Matrices: A Comparative Study in a Nonhuman Primate Model. Tissue Eng. Part A.

[91] Hiwatari K.I., Kajiwara A., Nakagawa K., Nakajima S., Fukushima K., Hironaka K., Kumagai H., Shigeno K. (2026). Oxygen-controlled gamma-irradiation and annealing enable terminal processing of collagen-based biomaterials. Biomater. Adv..

[92] Le L.T.T., Trinh T.T., Giang N.N., Zhang X.R., Jin Y.X., Lee Y.H., Shin Y.W., Bae J.W., Ngoc Chien P., Heo C.Y. (2025). Development and Evaluation of an Injectable Acellular Dermal Matrix for Breast Reconstruction. ACS Appl. Bio Mater..

[93] Zuo H., Peng D., Zheng B., Liu X., Wang Y., Wang L., Zhou X., Liu J. (2012). Regeneration of mature dermis by transplanted particulate acellular dermal matrix in a rat model of skin defect wound. J. Mater. Sci. Mater. Med..

[94] Cho J., Hwang H., Song S.Y., Suh H.P., Hong J.P. (2023). Evaluation of wound healing effects of micronized acellular dermal matrix in combination with negative pressure wound therapy: In vivo study. Int. Wound J..

[95] Jin H., Shin B., Kim D., Seo Y., Lee Y., Lee S.M. (2026). Ultra-fine micronized human dermal matrix reprograms the chronic pressure-ulcer microenvironment to accelerate healing. J. Appl. Biomater. Funct. Mater..

[96] Gabriel A., Gottenger R., Kabaria N., Huang L.T., Leamy P., Stefanelli V., Zuo W., Stec E., Gardocki-Sandor M., Xu H. (2026). Host Biologic Response to Acellular Dermal Matrices of Human and Porcine Origin. Aesthet. Surg. J. Open Forum.

[97] Biehl A., Gracioso Martins A.M., Davis Z.G., Sze D., Collins L., Mora-Navarro C., Fisher M.B., Freytes D.O. (2023). Towards a standardized multi-tissue decellularization protocol for the derivation of extracellular matrix materials. Biomater. Sci..

[98] van Hengel E.V.A., van der Laan L.J.W., de Jonge J., Verstegen M.M.A. (2025). Towards Safety and Regulation Criteria for Clinical Applications of Decellularized Organ-Derived Matrices. Bioengineering.

[99] Liu Y., Suarez-Arnedo A., Riley L., Miley T., Xia J., Segura T. (2023). Spatial Confinement Modulates Macrophage Response in Microporous Annealed Particle (MAP) Scaffolds. Adv. Healthc. Mater..

[100] Therriault M.A., Kottapalli S., Artsen A., Knight K., King G., Meyn L., Brown B.N., Moalli P.A. (2025). Profiling of the macrophage response to polypropylene mesh burden in vivo. Biomaterials.

[101] Park J.W., Choi S.Y., Kim K.R., Jin M., Seok J., Yoo K.H., Kim B.J. (2022). A randomized, participant- and evaluator-blinded, matched-pair prospective study to compare the safety and efficacy between polycaprolactone-based fillers in the correction of nasolabial folds. Dermatol. Ther..

[102] Signori R., Barbosa A.P., Cezar-Dos-Santos F., Carbone A.C., Ventura S., Nobre B.B.S., Neves M., Câmara-Souza M.B., Poluha R.L., De la Torre Canales G. (2024). Efficacy and Safety of Poly-l-Lactic Acid in Facial Aesthetics: A Systematic Review. Polymers.

[103] Ting W., Chong Y., Long X., Shu M., Wang H., Huang J., Zeng A., Bai Z., Wang R., Zhang X. (2024). A Randomized, Evaluator-Blinded, Multicenter Study to Compare Injectable Poly-D,L-Lactic Acid vs Hyaluronic Acid for Nasolabial Fold Augmentation. Aesthet. Surg. J..

[104] Lee Y.I., Chau N.H., Nguyen N.H., Ham S., Baek Y., Kim J., Lee J.H. (2026). Injectable Particulated Human Acellular Dermal Matrix Booster for Skin Restoration: An Integrated Randomized, Split-Face, Double-Blinded Clinical Trial and Preclinical Study. Int. J. Mol. Sci..

[105] Zhang C., Chang X., Chen L., Wang H., Feng G., Zheng W., Li L., Xu W., Qi X., Jiang X. (2026). Safety and Efficacy of Micronized Acellular Dermal Matrix Injection for Correction of Moderate to Severe Nasolabial Folds: A Double-Blind, Multicenter, Randomized Controlled, Non-inferior Clinical Trial. Aesthetic Plast. Surg..

[106] Zhang E., Wang Y., Zhou Q., Zheng B., Wu Q., Zhang Y., Chen A., Chen C. (2026). Effects of poly-L-lactic acid combined with acellular dermal matrix in the treatment of abdominal adiposity, skin laxity, and striae gravidarum. Am. J. Transl. Res..

[107] Chen S.Y., Chen S.T., Lin J.Y., Lin C.Y. (2020). Reconstitution of Injectable Poly-d,l-lactic Acid: Efficacy of Different Diluents and a New Accelerating Method. Plast. Reconstr. Surg. Glob. Open.

[108] Tao M., Ao T., Mao X., Yan X., Javed R., Hou W., Wang Y., Sun C., Lin S., Yu T. (2021). Sterilization and disinfection methods for decellularized matrix materials: Review, consideration and proposal. Bioact. Mater..

[109] Capella-Monsonís H., Crum R.J., Hussey G.S., Badylak S.F. (2024). Advances, challenges, and future directions in the clinical translation of ECM biomaterials for regenerative medicine applications. Adv. Drug Deliv. Rev..

