# Supplementary material for: Micronized Acellular Dermal Matrix and Synthetic Polymers: Temporal Macrophage Transition as a Mechanistic Framework
Source: Int J Mol Sci. 2026 Oct 18;27(20):9239. doi: 10.3390/ijms27209239 (PMC13705086; doi:10.3390/ijms27209239)
Supplement: Supplementary file 1 [file ijms-27-09239-s001.zip › ijms-4604443-supplementary.pdf]

Table S1. Previously reviewed domains, unresolved questions, and the specific contribution of the present review.

What has been reviewed	What remains unresolved	Contribution of the present review
Reviews of dECM/ADM and related composites have addressed material preparation, preservation of ECM composition and architecture, host interactions, and clinical translation [1,3,28].	How mADM-derived cues interact in space and time with persistent synthetic-polymer stimulation in standardized injectable co-formulations remains unclear.	Defines the material-specific question for polymer‑mADM systems and distinguishes direct evidence from ADM/mADM-containing systems from mechanistic extrapolation based on other ECM sources.
Reviews of injectable synthetic polymers and foreign-body responses have examined material persistence and degradation, immune responses, and neocollagenesis [2,5,11].	It remains uncertain how adding mADM modifies the magnitude and duration of polymer-associated stimulation and its relationship to macrophage trajectories and tissue remodeling.	Integrates polymer persistence and degradation with evolving mADM-derived cues and identifies component ratio, total dose, spatial distribution, and degradation/remodeling kinetics as testable formulation variables.
Macrophage heterogeneity, temporal changes in macrophage states, and their influence on tissue repair have been investigated [9,21,29].	Whether a population-level macrophage trajectory mediates potential polymer–mADM synergy has not been established, and no universal switching time has been defined.	Proposes a testable temporal framework, a functionally defined macrophage polarization window, and evidence criteria and falsifiable predictions for validating the proposed mechanism.

Note: Reference numbers correspond to the main manuscript. dECM, decellularized extracellular matrix; ADM, acellular dermal matrix; ECM, extracellular matrix; mADM, micronized acellular dermal matrix.
